# Ningxiang pig-derived *Enterococcus hirae* protects against *E. coli*-induced gut dysbiosis and inflammation via acetate/propionate-MyD88-NF-κB axis in piglets

**DOI:** 10.1186/s40168-025-02310-8

**Published:** 2026-01-06

**Authors:** Longlin Zhang, Zichen Wu, Haibo Shen, Yunlong Meng, Hongkun Li, Rong Cai, Dalin Tang, Meng Kang, Yulong Yin, Bie Tan, Jing Wang

**Affiliations:** 1 College of Animal Science and Technology, Key Laboratory for Quality Regulation of Livestock and Poultry Products of Hunan Province, Hunan Agricultural University, Changsha, 410128 China; 2 Yuelushan Laboratory, Changsha, 410128 China; 3 Laboratory of Animal Nutritional Physiology and Metabolic Process, Key Laboratory of Agroecological Processes in Subtropical Region, National Engineering Laboratory for Pollution Control and Waste Utilization in Livestock and Poultry Production, Institute of Subtropical Agriculture, Chinese Academy of Sciences, Changsha, 410125 China

**Keywords:** Intestinal inflammation, *E. hirae*, Ningxiang piglets, Acetate/propionate-MyD88-NF-κB axis

## Abstract

**Background:**

Intestinal inflammation, often driven by microbial dysbiosis and infections, remains a significant health challenge with limited effective treatments. Identifying probiotic strains with anti-inflammatory properties and elucidating their mechanisms is essential for developing novel therapeutic strategies. This study investigates the molecular mechanisms by which *E. hirae*—a lactic acid bacterium (LAB) isolated from Ningxiang piglets with low diarrhea incidence—alleviates *E. coli*-induced intestinal inflammation.

**Results:**

In the present study, comparative analysis showed that Ningxiang piglets exhibited a significantly lower incidence of diarrhea and reduced *E. coli* abundance compared to Yorkshire piglets. Notably, *E. hirae* was more abundant in Ningxiang piglets and correlated with elevated secretory IgA levels. Additionally, in vitro antagonism assays found that *E. hirae* effectively inhibited E. coli growth. In vivo supplementation of *E. hirae* in *E. coli*-infected piglets restored intestinal microbial balance, increased levels of short-chain fatty acids (SCFAs) such as acetate and propionate, and mitigated *E. coli* colonization. Further analyses suggested that acetate and propionate downregulated the MyD88/NF-κB signaling pathway, thereby reducing pro-inflammatory cytokine expression. Molecular docking and MyD88 ^− / − ^experiments verified that MyD88 is involved in SCFA-mediated protection against *E. coli*-induced inflammation. Furthermore, analyses of public human datasets revealed that Crohn’s disease patients exhibited a similar reduction in SCFA levels and MyD88–NF-κB pathway activation, suggesting potential clinical relevance.

**Conclusion:**

Token together, our results reveal that Ningxiang pig-derived *E. hirae* alleviates *E. coli*-induced gut dysbiosis and inflammation potentially through the acetate/propionate–MyD88–NF-κB axis. This work provides mechanistic insights for further exploration of probiotic and postbiotic approaches against bacterial-induced intestinal inflammation.

Video Abstract

**Supplementary Information:**

The online version contains supplementary material available at 10.1186/s40168-025-02310-8.

## Introduction

Intestinal inflammation adversely affects host health and can be triggered by various factors, including microbial dysbiosis, dietary influences, and immune dysfunction [1–3]. Among inflammatory bowel diseases (IBDs), such as Crohn’s disease (CD), gut microbiota dysbiosis is closely associated with disease onset and progression [4–6]. Growing evidence highlights the role of *Escherichia coli* (*E. coli*) in intestinal inflammation, as its increased abundance is frequently observed in patients with CD [7–12]. As a mucosa-associated bacterium, *E. coli* is highly susceptible to adhering to small intestinal epithelial cells, secreting enterotoxins, causing intestinal barrier dysfunction, promoting the expression of inflammatory factors, and triggering intestinal inflammation [13]. Therefore, it is necessary to develop effective strategies to inhibit bacterial proliferation and enterotoxin production to attenuate the occurrence of *E. coli*-associated intestinal inflammation.

Probiotics have emerged as promising approaches for preventing and alleviating intestinal inflammation [14–16]. Lactic acid bacteria (LAB), as important probiotics in the healthy intestinal microbiota, contribute to regulating gut development and maintaining mucosal barrier function through early colonization [17–19]. In recent years, probiotic research has gradually shifted its focus toward postbiotics–metabolic products of probiotics such as SCFAs [20–22], peptidoglycan [23–25], and bacteriocins [26–28]–that have garnered attention for their potential health-promoting effects, including immunomodulation, anti-inflammation, antimicrobial properties, and gut barrier protection. However, the bioactivity and precise mechanisms of these postbiotics remain complex and are yet to be fully elucidated.

Although rodent models are useful for molecular and cellular studies, they do not fully reflect the physiological and pathological characteristics of the human gut. In contrast, pigs, with gut microbiota composition and immune responses closely resembling those of humans, serve as a suitable model for studying gut biology and dietary interventions [29–31]. Their susceptibility to *E. coli* infections, due to similar intestinal receptors and immune responses, makes them valuable for research on gastrointestinal diseases and host-microbe interactions [32, 33].

In this study, we compared Ningxiang and Yorkshire piglets at different postnatal stages and found that Ningxiang piglets had a lower incidence of diarrhea and a reduced *E. coli* colonization rate. Additionally, their gut microbiota was enriched with *Enterococcus hirae* (*E. hirae*), which showed a strong correlation with sIgA levels. Using *E. coli*-challenged piglet and cell models, we systematically evaluated the anti-inflammatory effects of *E. hirae*. The results demonstrated that *E. hirae* effectively inhibited *E. coli* colonization, restored gut microbiota balance, promoted acetate and propionate production, and mitigated inflammation by suppressing the aberrant activation of the MyD88-NF-κB signaling pathway, reducing pro-inflammatory cytokines, and enhancing intestinal barrier integrity. Importantly, reductions in these SCFAs and activation of the MyD88-NF-κB pathway were also observed in patients with CD. Taken together, our study elucidates a mechanism by which Ningxiang pig-derived *E. hirae* and its postbiotic metabolites alleviate *E. coli*-induced intestinal inflammation, providing a foundation for exploring their potential in addressing intestinal inflammatory disorders.

## Materials and methods

### Bacterial strain

The *E. hirae* strain used in this study was originally isolated from the feces of healthy Ningxiang piglets using selective enrichment in MRS agar plates, which were cultured anaerobically in MRS medium at 37 °C for 18 h. *Escherichia coli* K88, provided by Prof. Wenkai Ren (South China Agricultural University), was grown aerobically in LB broth at 37 °C.

### Animal experiments

In the mouse model of *E. hirae* treatment, 6-week-old male C57BL/6 mice were housed and maintained in SPF conditions. The mice were administered *E. hirae* (10^8^ CFU) or sterile saline for 3 weeks by oral gavage. After three weeks, the mice were sacrificed by cervical dislocation, and serum, intestine, and fecal samples were collected for further analysis.

In the mouse model of acetate and propionate treatment, 6-week-old male C57BL/6 mice were housed and maintained in SPF conditions. Sodium acetate (Sigma, #S5636) or sodium propionate (Sigma, #P1880) (200 mM each) was provided to SPF C57BL/6 J mice in drinking water for 33 days, and then mice were treated with 10^8^ CFU *E. coli* on day 30. After 33 days, mice were sacrificed by cervical dislocation, and serum, intestine, and fecal samples were collected for further analysis.

In the Myd88^−/−^ mouse model, 6-week-old C57BL/6 mice were housed and maintained in SPF conditions. Sodium acetate (Sigma, #S5636) or sodium propionate (Sigma, #P1880) (200 mM each) was provided to Myd88^f/f^mice in drinking water for 33 days, and then the mice were treated with 10^10^CFU*E. coli* at day 30. Additionally, Myd88^f/f^ and Myd88^−/−^ mice were provided with water for 33 days, and then Myd88^−/−^ mice were treated with 10^8^CFU*E. coli* at day 30. After 33 days, all mice were sacrificed by cervical dislocation, and serum, intestine, and fecal samples were collected for further analysis.

In the cells model, the IPEC-J2 intestinal epithelial cell line was cultured in DMEM/F12 medium supplemented with 100 U/mL penicillin, 100 mg/mL streptomycin, and 10% (v/v) FBS, under standard conditions (37 °C, 5% CO2, humidified atmosphere). In the *E. hirae* co-culture group, cells were first incubated with live *E. hirae* (1 × 10^6^ CFU/well) for 2 h, followed by exposure to *E. coli* (1 × 10^5^ CFU/well) for 1 h. For SCFAs pretreatment, cells were treated with a 10 mM acetate/propionate mixture for 24 h prior to a 1 h *E. coli* challenge (1 × 10^5^ CFU/well). All treatments were performed in triplicate.

In the two breeds of piglets, a total of 179 litters of Yorkshire pigs and 138 litters of Ningxiang pigs from both breeds were recorded for diarrhea rate analysis. Fecal consistency was scored daily using a standardized scale: 0 (normal), 1 (mild), 2 (moderate), or 3 (severe diarrhea). Fecal samples were collected from 22 Ningxiang piglets and 26 Yorkshire piglets on day 7. The study was conducted at the Yongxing Pig Farm of Hunan Chuweixiang Agriculture and Animal Husbandry Co., Ltd. (Changsha, China) and at Sifanghong farm (ZhangJiakou, China).

In the pig model of *E. coli*-induced intestinal inflammation, twenty-four-day-old DLY piglets with comparable initial body weights were stratified into three groups: (1) CON group: Standard basal diet + daily oral administration of 10 mL sterile saline (days 0–17). (2) Enterotoxigenic *Escherichia coli* (ETEC) group: Standard diet + saline gavage (days 0–17) followed by 10^10^ CFU *E. coli* challenge via gastric infusion (days 15–17). (3) Eh + ETEC group: Standard diet + daily 10^10^ CFU *E. hirae* suspended in 10 mL saline (days 0–17) with concurrent *E. coli* challenge (days 15–17).

### Histopathology and goblet cell staining

Ileal tissues were fixed in 10% neutral-buffered formalin for 24 h, paraffin-embedded, and sectioned (10 µm), as previously reported [34, 35]. Sections were stained with hematoxylin and eosin (H&E). Villus height and crypt depth were quantified using ImageJ v1.53. Additionally, mucin-producing goblet cells were visualized using Alcian Blue (pH 2.5)/Periodic Acid-Schiff (AB-PAS) staining (Abcam #ab150662). Sections were imaged under an Olympus BX53 microscope.

### Inflammatory cytokine and intestinal barrier biomarkers

Quantification of cytokine levels in piglets ileal mucosa was assessed using a commercially available porcine cytokine multiplex immunoassay kit (RayBiotech, Norcross, GA). The concentration of cytokines was calculated by QAP-CYT-1 software. Additionally, the concentrations of sIgA in serum were determined by ELISA assay (piglets sample:CSB-E12063p, mouse sample: CSB-E08413m, CUSABIO, https://www.cusabio.com/). And the concentrations of DAO and D-lactate in all serum samples were also determined by ELISA assay (Ruixinbio Quanzhou, China). Meanwhile, the content of ET in all serum samples was tested by commercial Lipopolysaccharide limulus test kit (Xiamen Bioendo Technology Co., Ltd., Xiamen China).

### Immunofluorescence and quantification

Ileum tissues were fixed in 4% paraformaldehyde, cryoprotected in 30% sucrose, and embedded in OCT. Frozen Sects. (10 µm) were permeabilized with 0.1% Triton X-100, blocked with 5% BSA, and incubated overnight with anti-MyD88 (Proteintech #23,230–1-AP, 1:200) or anti-PCNA (Abcam #ab92552, 1:500) antibodies. Alexa Fluor 488–conjugated secondary antibodies (Thermo Fisher #A-11008, 1:1000) were applied, and nuclei were counterstained with DAPI. Images were acquired using a Zeiss LSM 880 confocal microscope.

### RT-PCR

Total RNA was extracted using the RNeasy Plus Mini Kitb (AG21024). cDNA synthesis utilized the PrimeScript RT Reagent Kit (AG11728). Quantitative PCR was performed on a QuantStudio 6 Flex System (Applied Biosystems) with SYBR Green Master Mix (AG11701). All the reagents are purchased from ACCURATE BIOTECHNOLOGY(HUNAN) CO., LTD (ChangSha, China). The primers used are listed in Supplementary Table S1.

### Western blot analysis

Frozen intestine tissue samples were homogenized in RIPA lysis buffer (Beyotime, China), supplemented with a cocktail of protease inhibitors (Beyotime, China). The lysates were collected and then heated in boiled water for approximately 5 min. Equal amounts of protein samples were subjected to 10% sodium dodecyl sulfate–polyacrylamide gel electrophoresis gels and separated under proper electro-transferred conditions. These target proteins were transferred onto polyvinylidene fluoride membranes. Membranes were blocked in 5% skimmed milk for 2 h at RT, washed in TBST buffer, and incubated overnight in primary antibodies at 4 ◦C and secondary antibodies (anti‐rabbit IgG; Servicebio; GB21404). The primary antibodies TLR4 (19,811–1-AP), p-p65 (82,335–1-RR), Myd88 (67,969–1-Ig) were purchased from Proteintech. P65 (L8F6) was purchased from Cell Signaling Technology. MCT1 (AWA46364) was purchased from Abiowell. β-actin (Em21002) was purchased from HUABio. Thereafter, the bands were washed in TBST and were then exposed to X-ray film. Protein blots were quantified by Image-Pro Plus (version 6.0), normalized to β-actin.

### SCFAs profiling

The concentration of SCFAs in fecal and ileal samples was derived with N-tert-butyldimethylsilyl-N-methyltrifluoroacetamide (MTBSTFA) and analyzed via gas chromatography (GC, Agilent 8890) equipped with a DB-FFAP column (30 m × 0.25 mm × 0.25 µm). Calibration curves were generated using pure acetate, propionate, butyrate, and valerate standards (Sigma). All procedures in GC were performed in triplicate.

### *E. coli* quantification

DNA was extracted using the FastDNA Stool Kit (SENO Biotech Co., Ltd., Zhangjiakou, China).* E. coli* levels were determined by qPCR on a LightCycler 480 II (Roche). The absolute abundance of *E. coli* was quantified by extrapolating cycle threshold (Ct) values to a standard curve and expressed as logarithmic gene copy numbers normalized to total DNA content in ileal digesta and fecal samples [36]. A standard curve was created using serial tenfold dilutions of 413 bp pure DNA corresponding to 10^2^ to 10^10^ gene fragment numbers (using 96 bp as primer). The primers used are listed in Supplementary Table S1.

### 16s rRNA gene sequencing

DNA was extracted from colonic contents using a Stool DNA Isolation Kit (SENO Biotech Co., Ltd., Zhangjiakou, China). The V3–V4 region of the 16S rRNA gene was amplified using the universal primer pair 338 F (5’–ACTCCTACGGGAGGCAGCA–3’) and 806R (5’–GGACTACHVGGGTWTCTAAT–3’), with Illumina sample-specific indices attached for sequencing. PCR amplicons were purified, quantified, and pooled for sequencing on an Illumina NovaSeq 6000 platform. Illumina sequencing and processing of sequencing data were performed by Beijing Biomaker Technology Co., Ltd. (China).

### Modularity network analysis

A modularity network analysis was conducted to explore the relationships between bacterial taxa. Relative abundance data at the genus level were used to construct correlation networks. The data were filtered to remove OTUs with zero abundance across all samples, and Spearman correlation was computed using the corr.test() function in the psych package with false discovery rate (FDR) correction. Only correlations with an absolute correlation coefficient (|r|) ≥ 0.6 and *p*-value ≤ 0.05 were retained for network construction. The filtered correlation data were used to generate a graph structure using the igraph package. Community detection was performed using the walktrap algorithm to identify densely connected subgraphs, representing modular groups within the network. Each node’s degree (number of connections) was calculated to identify hub taxa, and nodes were colored based on their community assignment. Nodes and edges were visualized using the ggraph package, with the Fruchterman–Reingold layout employed to display the network structure. The final network included only major communities with at least 10 nodes. Nodes with the highest degree were highlighted to indicate potential keystone taxa.

### FISH assay

A Cy3-labeled rRNA probe specific to the 16S rRNA sequence of *E. coli* was used for FISH (eco1167: 5’– GCATAAGCGTCGCTGCCG–3’). Piglets and mouse intestine samples were fixed with 4% PFA at 4 °C for 12 h, then treated with cold 96% ethanol (1:1 v/v) for fixation. The samples were dehydrated in ethanol (50%, 80%, 96%) and dried at 46 °C for 3 min. Permeabilization was done with lysozyme (2 mg/mL) at 37 °C for 1 h. After drying, hybridization was carried out at 46 °C for 2 h with a buffer containing 5 M NaCl, 1 M Tris–HCl, SDS, formamide, and the Cy3-labeled probe. Samples were then washed with a buffer at 48 °C for 10 min and briefly dipped in ice-cold ddH2O. Finally, the tissues were placed on slides and analyzed with an EVOS imaging system and a confocal microscope.

### Whole-genome analysis of *E. hirae*

PacBio sequencing data was processed using the SMRT Link tool to generate Circular Consensus Sequencing (CCS) reads. Reads shorter than 2,000 bp were filtered out to obtain high-quality sequences for downstream analysis. The filtered CCS reads were assembled using Hifiasm software. The assembly was further corrected using Pilon software with Illumina data to improve accuracy. Assembled contigs were aligned against the NT database to determine the chromosome type. All processes were performed by biomarker Technologies (www.biomarker.com.cn) and BMKcloud (www.biocloud.net).

### Genomic circos plot and KEGG analysis of *E. hirae*

The assembled genome and annotated features, including tRNA, rRNA, repeat sequences, GC content, and gene functional categories, were visualized using Circos v0.66. The Circos plot provides a clear view of genomic components and their relationships. The plot includes several concentric rings: genome size, genes on positive and negative strands with COG functional categories, repeat sequences, tRNA (blue) and rRNA (purple), GC content (yellow for higher, blue for lower than average), and GC skew (dark gray for G > C, red for C > G). Additionally, KEGG (Kyoto Encyclopedia of Genes and Genomes) pathway analysis was performed to annotate gene functions and identify metabolic pathways in *E. hirae* HNAU0516. Genes were mapped to KEGG pathways to assess their involvement in various biological processes, with a particular focus on carbohydrate metabolism pathways. This analysis helped to identify key functional capabilities of the strain, providing insights into its metabolic potential.

### TEM preparation method for *E. hirae*

Transmission Electron Microscopy (TEM) was used to visualize *E. hirae*. Bacterial cells were fixed with TEM fixative and post-fixed in osmium tetroxide. After dehydration in an ethanol series, samples were embedded in 812 resin, polymerized, and sectioned into 60–80 nm ultrathin slices. Sections were stained with uranyl acetate and lead citrate, and imaged using a Hitachi HT7800 transmission electron microscope.

### In vitro tolerance assay in artificial intestinal juice

Artificial intestinal juice was prepared based on the guidelines from the Chinese Pharmacopoeia (2020 edition). Artificial intestinal juice was prepared by dissolving 5.59 g of K₂HPO₄ and 0.41 g of KH₂PO₄ in 1,000 mL of deionized water, adjusting the pH to 8.0, and adding 1 g of pancreatin per 100 mL of solution. The solution was filtered through a 0.22 μm membrane and stored at 4 °C. To evaluate artificial intestinal juice tolerance, *E. hirae* was first incubated in artificial intestinal juice (pH 8.0) at 37 °C for either 0 h or 1 h. After the designated incubation period, the strain was inoculated onto MRS agar plates for growth, and CFU were counted to determine survival rates. All experiments were conducted in triplicate.

### Bile tolerance

Bile salt tolerance was assessed by preparing bile solutions of different concentrations (0.03%, 0.1%, 0.2%, 0.3%, 0.4%, and 0.5%) through serial dilution of a 10% bile salt stock solution sterilized at 121 °C for 20 min. The *E. hirae* strain was first incubated in MRS broth supplemented with bile salt concentrations of 0.3% or 0.4% (w/v) at 37 °C for either 0 h or 1 h. After the specified incubation period, *E. hirae* was inoculated onto MRS agar plates, and the number of colony-forming units (CFU) was determined to assess the survival rate. All experiments were conducted in triplicate.

### Antibiotic sensitivity analysis

The antibiotic sensitivity of *E. hirae* was evaluated using a panel of commonly used antibiotics, including penicillin, streptomycin, vancomycin, rifampicin, kanamycin, erythromycin, ampicillin, gentamicin, tetracycline, ciprofloxacin, chloramphenicol, and clindamycin. The *E. hirae* strain was inoculated onto MRS agar plates containing each of the antibiotics, and growth was assessed after incubation to determine susceptibility or resistance to each antibiotic.

### Biochemical identification of carbohydrate metabolism

The carbohydrate metabolism capacity of *E. hirae* was determined using biochemical identification tubes to evaluate the strain’s ability to utilize various polysaccharides and carbohydrates. The tested carbohydrates included lactose, raffinose, maltose, salicin, cellobiose, hesperidin, serum inulin, sucrose, sodium hippurate, sorbitol, and mannitol. The utilization of each carbohydrate was indicated by a color change in the test tubes, and results were compared to an uninoculated control.

### Dynamic molecular docking

The acetate and propionate were docked to the MyD88 protein using AutoDock Vina 1.1.2. The three-dimensional structures of acetate (compound CID: 175) and propionate (compound CID: 1032) were retrieved from the PubChem database (https://pubchem.ncbi.nlm.nih.gov), and converted from SDF to PDB format using Open Babel 2.3.2. The protein structure of MyD88 (Protein Data Bank ID: 4DOM) was obtained from the RCSB Protein Data Bank (www.rcsb.org). The protein structure was prepared using PyMOL 2.3.4 to remove water molecules and ligands, followed by further modifications such as hydrogen addition and charge balancing using AutoDock Tools. Both the receptor protein and ligands were then converted to PDBQT format. The docking process was performed using AutoDock Vina 1.1.2, with a grid box generated using AutoDock Tools to define the binding region. All relevant torsion angles of the ligands were treated as rotatable to allow for the exploration of conformational space. A total of 100 docking poses were generated for each ligand, and the pose with the lowest binding free energy was selected as the optimal pose. The docking results were analyzed using PLIP to identify key interactions between the ligands and the receptor. Visualizations of the docking results were generated using PyMOL.

### Public dataset retrieval and analysis for CD cohort

Untargeted metabolic data for SCFA concentrations in human stools (including non-IBDs, CD patients) was obtained from Project ST000923 and ST001000 in the Metabolomics Workbench (http://www.metabolomicsworkbench.org). The correlation of CD and SCFAs (propionate, butyrate, and butyrate/isobutyrate) was analyzed by linear regression. Additionally, bulk RNA-sequencing data for biopsies collected from the ileum are accessed through GEO Series accession number GSE193677 and GSE137344.

### Statistical analysis

The data were analyzed using SPSS 26.0 statistical software (ver. 26.0 for Windows, SPSS Inc., Chicago, IL, USA) and R v4.3.3 within RStudio v1.4.1717. Data were expressed as means with their standard errors. A two-tailed Student’s t test was used to detect statistical significance for two groups, while one-way ANOVA with two-tailed t-tests was used for more than two groups. Statistical significance was defined as *p* < 0.05.

## Results

### The microbial biomarker in stool samples of suckling piglets with low diarrhea is significantly correlated with sIgA

**Fig. 1:**
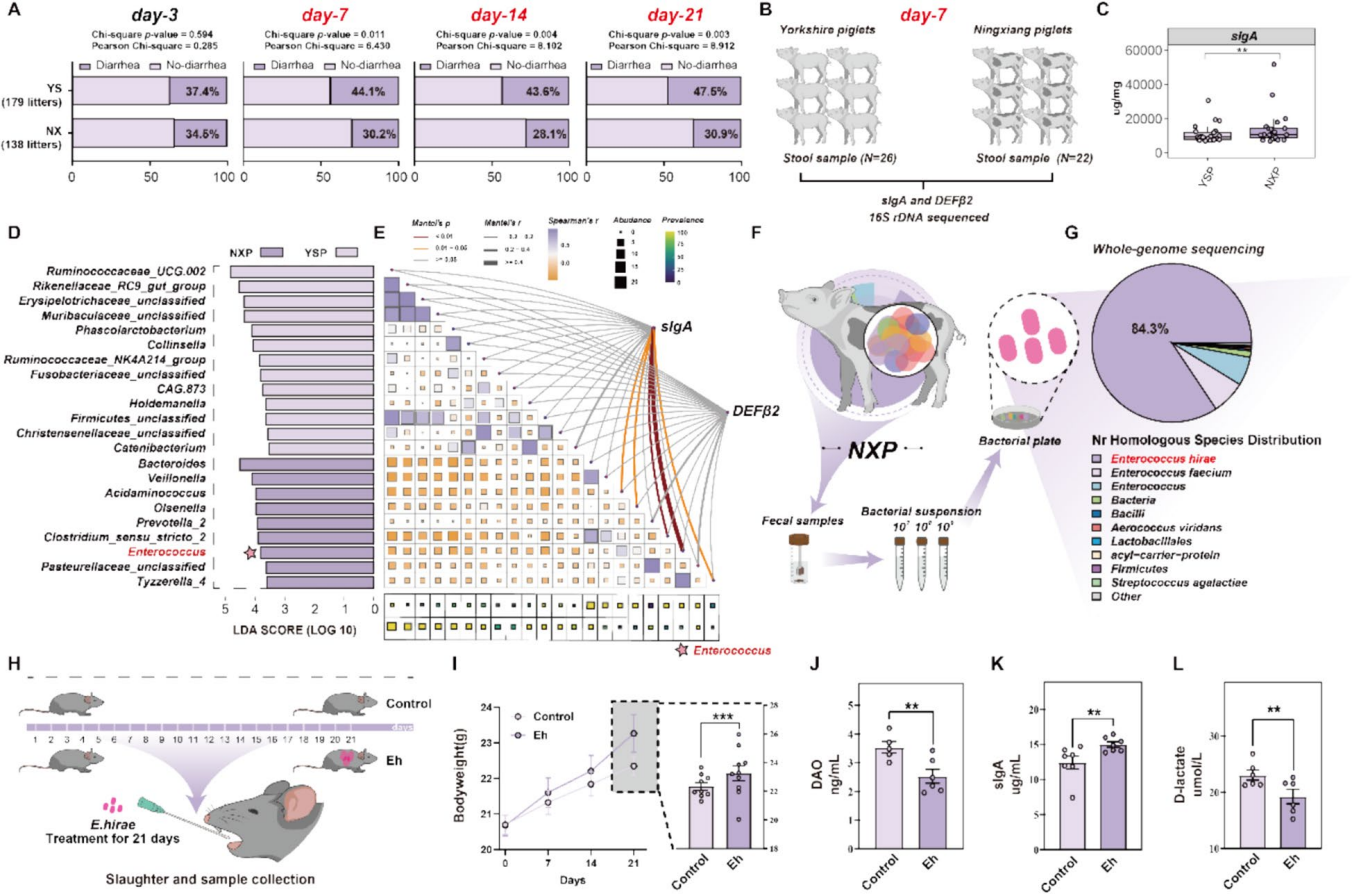
The microbial biomarker in stool samples of suckling piglets with low diarrhea is significantly correlated with sIgA. **A** The diarrhea incidence during the suckling period (day-3, day-7, day-14 and day-21) between Ningxiang pig (138 litters) and Yorkshire pig (179 litters). **B** Schematic depicting fecal samples collection from two breeds piglets at day-7. **C** The result of sIgA concentration in two breeds piglets stool samples at day-7. **D** Identification of the most differentially abundant microbes among two breeds. The plots were generated from LEFSe analysis with LDA scores above 3.5 and *P*-values below 0.05. **E** The heatmap demonstrates the Spearman correlation and prevalence between two breed piglets of significantly different bacteria, and the network diagram further demonstrates the correlation between sIgA and DEFβ2 with these bacteria, as assessed by the Mantel test. *p*-values and correlation coefficient *R*-values of the Mantel test are indicated by different line colors and thicknesses, respectively, while the Spearman correlation and prevalence are demonstrated by the color bars. **F** Schematic depicting culture-based techniques to isolate microbes from the collected Ningxiang piglet fecal suspensions. **G** Distribution of species based on sequences aligned to the Nr database after whole genome sequencing. **H** Schematic diagram of oral *E. hirae* in mice. **I** The results of body weight. **J**-**L** The concentration of DAO (**J**), sIgA (**K**), and D-lactate (**L**) in the mice serum samples

Initially, we observed that suckling Ningxiang piglets (138 litters) have stronger resistance to diarrhea than suckling Yorkshire piglets (179 litters) between day 7 and day 21 (day 3: 34.5% vs 37.4%; day 7: 30.2% vs 44.1%; day 14: 28.1% vs 43.6%; day 21: 30.9% vs 47.5%; respectively) (Fig. 1A). Consequently, stool samples were collected on day 7 from both breeds, with one piglet randomly selected from each litter (Fig. 1B). Firstly, the results showed that the sIgA concentration in the stool samples of Ningxiang piglets was significantly higher than that of Yorkshire piglets, although there was no difference in the concentration of DEFβ2 between the two breeds (Fig. 1C, Figure S1A).

We further analyzed the gut microbiota composition of both piglet breeds. A principal coordinates analysis (PCoA) based on Bray–Curtis was used to investigate the overall structure of stool gut microbiota and showed a separation in the gut microbiota structure among Ningxiang piglets and Yorkshire piglets (Figure S1F). At the phylum level, the relative abundances of Bacteroidetes, Proteobacteria, and Fusobacteria in Yorkshire piglets were significantly lower than those in Ningxiang piglets. At the same time, Firmicutes showed significantly higher relative abundance in the Yorkshire piglets (Figure S1G). At the genus level, the relative abundance of the top 15 most abundant communities was depicted in Figure S1H. *Lactobacillus* dominates the samples in both breeds. Additionally, *Fusobacterium* and *Bacteroides* had a significantly higher relative abundance in the Ningxiang piglets, while *Muribaculacaceae_unclassified* and *Ruminococcacear_UCG-002* had higher relative abundances in the Yorkshire piglets (Figure S1H). To ascertain candidate gut microbes potentially linked to diarrhea resistance, we employed linear discriminant analysis effect size (LEfSe) analysis, which revealed 9 differentially abundant gut microbes (LDA > 3.5) (Fig. 1D). Interestingly, we found that *Enterococcus* showed a strong correlation with sIgA levels in Ningxiang piglets (*p* < 0.01, R2 = 0.651) (Fig. 1E). Meanwhile, its prevalence rate in the stool samples of Ningxiang piglets was close to 100% (Fig. 1E). Taken together, these results suggest that *Enterococcus* is associated with low-diarrhea incidence in Ningxiang piglets and correlates with sIgA levels.

### Isolation of *Enterococcus. spp* and its probiotic potential

Studies have indicated that microbiota from different host sources may exhibit distinct genotypes, metabolic functions, and adaptive characteristics [37, 38]. To explore the unique beneficial gut microbiota features of Ningxiang piglets, we isolated *Enterococcus spp.* from suckling Ningxiang piglet fecal suspensions (Fig. 1F). Among the isolated microbes, we identified a strain, which was confirmed through whole-genome sequencing designated as *E. hirae* (Fig. 1G). Firstly, we found that *E. hirae* exhibits a uniform spherical shape with smooth and intact cell morphology (Figure S2A), and it can utilize various polysaccharide carbohydrates (Figure S2C-D), indicating its potential for survival and colonization in the gut. Additionally, *E. hirae* demonstrated resilience to simulated gastrointestinal conditions and high bile salt concentrations (Figure S2B), suggesting its capability to persist within the gastrointestinal tract and exert beneficial effects. Furthermore, we used antibiotic sensitivity tests and found that *E. hirae* was controllable, which is a key criterion for determining feasible probiotic candidates (Figure S2E).

To further determine its probiotic characteristics, we used a mouse model to explore its impact on host gut health (Fig. 1H). The results showed that oral administration of Ningxiang pig-derived *E. hirae* significantly increased the body weight of mice (F ig. 1I), small intestinal villus length, and crypt depth in both the jejunum and colon (Figure S3A-E). Additionally, it also significantly reduced the levels of serum DAO and D-LA, and increased the levels of serum sIgA (Fig. 1J-L).

### Ningxiang pig-derived *E. hirae* inhibits the colonization of *E. coli* and ameliorates intestinal inflammation

**Fig. 2:**
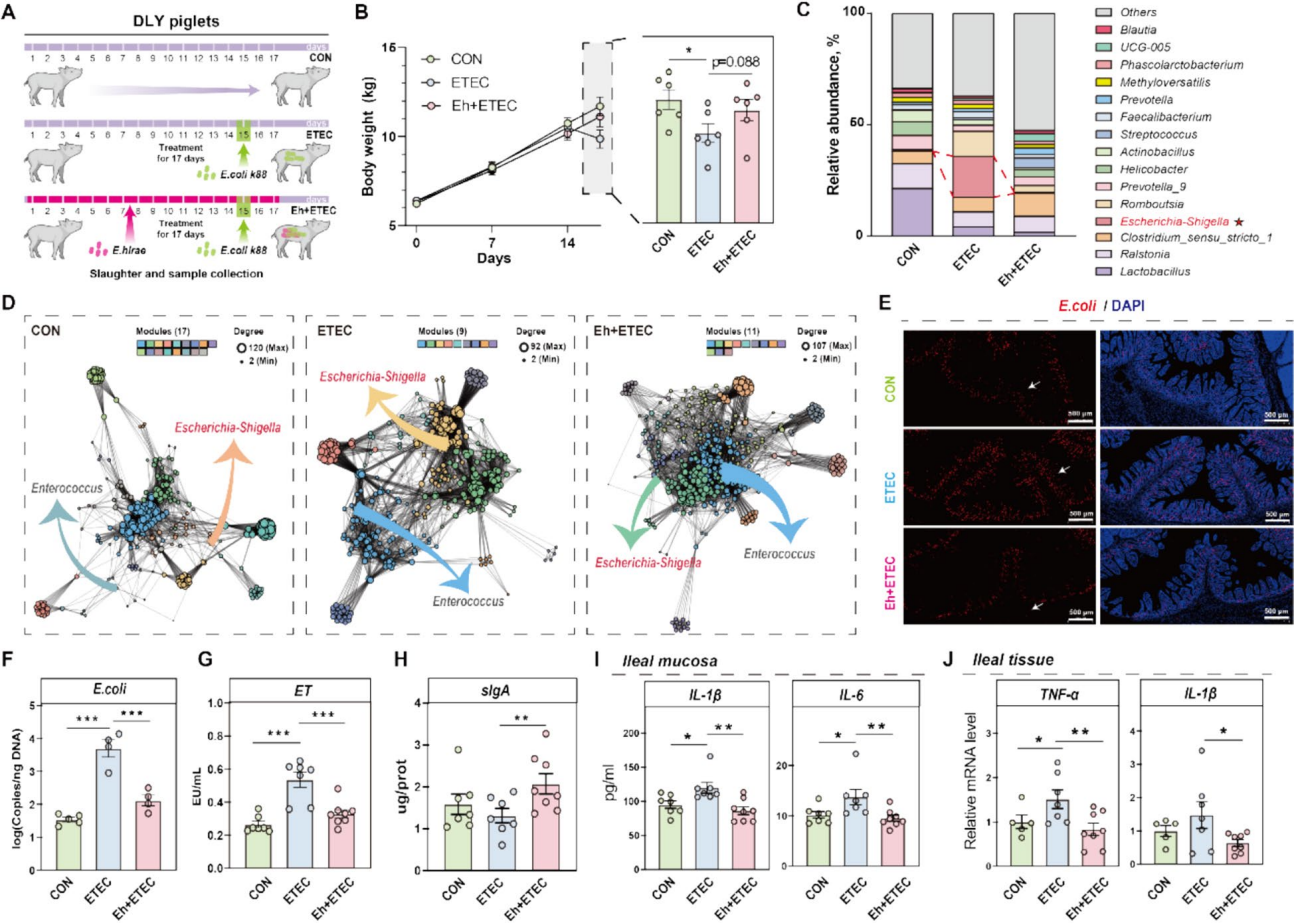
*E. hirae* inhibits the colonization of *E.coli* and ameliorates intestinal inflammation. **A** Schematic diagram of oral *E. hirae* in *E.coli*-treated piglet. **B** The result of body weight. **C** Comparisons of the relative abundance of ileum mucosal bacterial communities of piglets. The relative abundance was sorted, and only the top 15 genera are plotted, with the rest going to “Others”. **D** Network diagram with nodes colored according to each of ecological clusters (modules) in CON group, ETEC group and Eh + ETEC groups. The color of the arrows pointing to *Escherichia-Shigella* and *Enterococcus* are based on the color of the module in which they are located. **E** FISH analysis of *E. coli* in the ileum of CON group, ETEC group and Eh + ETEC groups. Colonized *E. coli* were detected by FISH with Cy3-labeled, rRNA-targeted oligonucleotide probes. **F** The *E. coli* content in piglets ileal digesta. **G** The concentration of ET in the piglet serum samples. **H** The concentration of sIgA in the piglet ileal digesta. **I** The concentration of IL-1β and IL-6 in the ileal mucosa. **J** The relative gene expression of TNF-α and IL-1β in the ileal tissue

*E. coli* and *Salmonella* are the primary pathogens causing diarrhea [39]. Firstly, we found that Ningxiang pig-derived *E. hirae* effectively inhibited *E. coli*, *Salmonella*, and *Staphylococcus aureus*, and its inhibitory effect on *E. coli* was the most obvious by using in vitro antibacterial assays (Figure S4A). Notably, the *E. coli* content in the feces of Yorkshire piglets was significantly higher than that in Ningxiang piglets at day 7 (Fig. 2B), leading us to hypothesize that *E. hirae* may serve as a key gut microbe for inhibiting *E. coli* in vivo. To validate this hypothesis, we conducted an animal experiment in which piglets were challenged with *E. coli* and orally administered Ningxiang pig-derived *E. hirae* for 17 days (Fig. 2A). As shown in Fig. 2B, *E. coli*-treated piglets exhibited a significant decrease in body weight, which was reversed upon administration of Ningxiang pig-derived *E. hirae*. We further explored the effect of *E. hirae* on the gut microbiota of *E. coli*-treated piglets using 16S-rDNA sequencing. As shown in Figure S5A-B, there was a significant difference in the α-diversity and β-diversity of the microbiota among the three experimental groups: the control (CON) group, the ETEC-challenged (ETEC) group, and the ETEC-challenged with *E. hirae* intervention (Eh + ETEC) group (Figure S5A-B). At the phylum level, the relative abundance of Proteobacteria was highest in the ETEC group, whereas it was lowest in the Eh + ETEC group (Figure S5C). At the genus level, the relative abundance of *Clostridium_sensu_stricto_1*, *Prevotella_9*, and *Prevotella* was higher in the Eh + ETEC group compared to the ETEC group (Fig. 2F). We found that the relative abundance of *Escherichia-Shigella* was significantly elevated in the ETEC group, while it remained significantly lower in the Eh + ETEC group (Fig. 2F). Furthermore, the results also showed that *E. hirae* intervention disrupted the dominance of *Escherichia-Shigella* and reduced its colonization (Fig. 2D). Compared to the CON group, *E. coli* infection significantly altered the microbial community network structure, reducing the number of modules and enhancing the dominance of *Escherichia-Shigella*. However, in the presence of *E. hirae* intervention, some community structures were restored, the ecological status of *Enterococcus* was enhanced, and the number of network modules increased, indicating that *E. hirae* improved the stability of the microbial community disrupted by ETEC (Fig. 2D). FISH staining and *E. coli* quantification further confirmed that *E. hirae* markedly inhibited the colonization of *E. coli* (Fig. 2E-F). The release of enterotoxin (ET), a key substance by ETEC [13], increased significantly in the serum of *E. coli*-treated piglets, while serum ET levels in the Eh + ETEC group were significantly reduced (Fig. 2G).

*E. coli* infection is known to readily induce intestinal inflammation and barrier dysfunction [13, 40]. In our results, orally administered Ningxiang pig-derived *E. hirae* significantly reversed the decrease of ileal sIgA (Fig. 2H), increased serum DAO and D-lactate (Figure S6C-D). Additionally, *E. hirae* significantly attenuated intestinal inflammation by decreasing pro-inflammatory cytokines IL-1β and IL-6 in the ileal mucosa of *E. coli*-treated piglets (Fig. 2I, Figure S6A). Meanwhile, in the ileal tissues of piglets treated with *E.coli*, the mRNA expression of pro-inflammatory cytokines TNF-α and IL-1β genes was also reduced by the administration of Ningxiang pig-derived *E. hirae* (Fig. 2J, Figure S6B). Additionally, *E. hirae* significantly alleviated intestinal damage and reversed the inhibition of PCNA ^+ ^protein induced by *E. coli* (Figure S6E-J).

### Acetate and propionate are the functional metabolites produced by Ningxiang pig-derived *E. hirae*

**Fig. 3:**
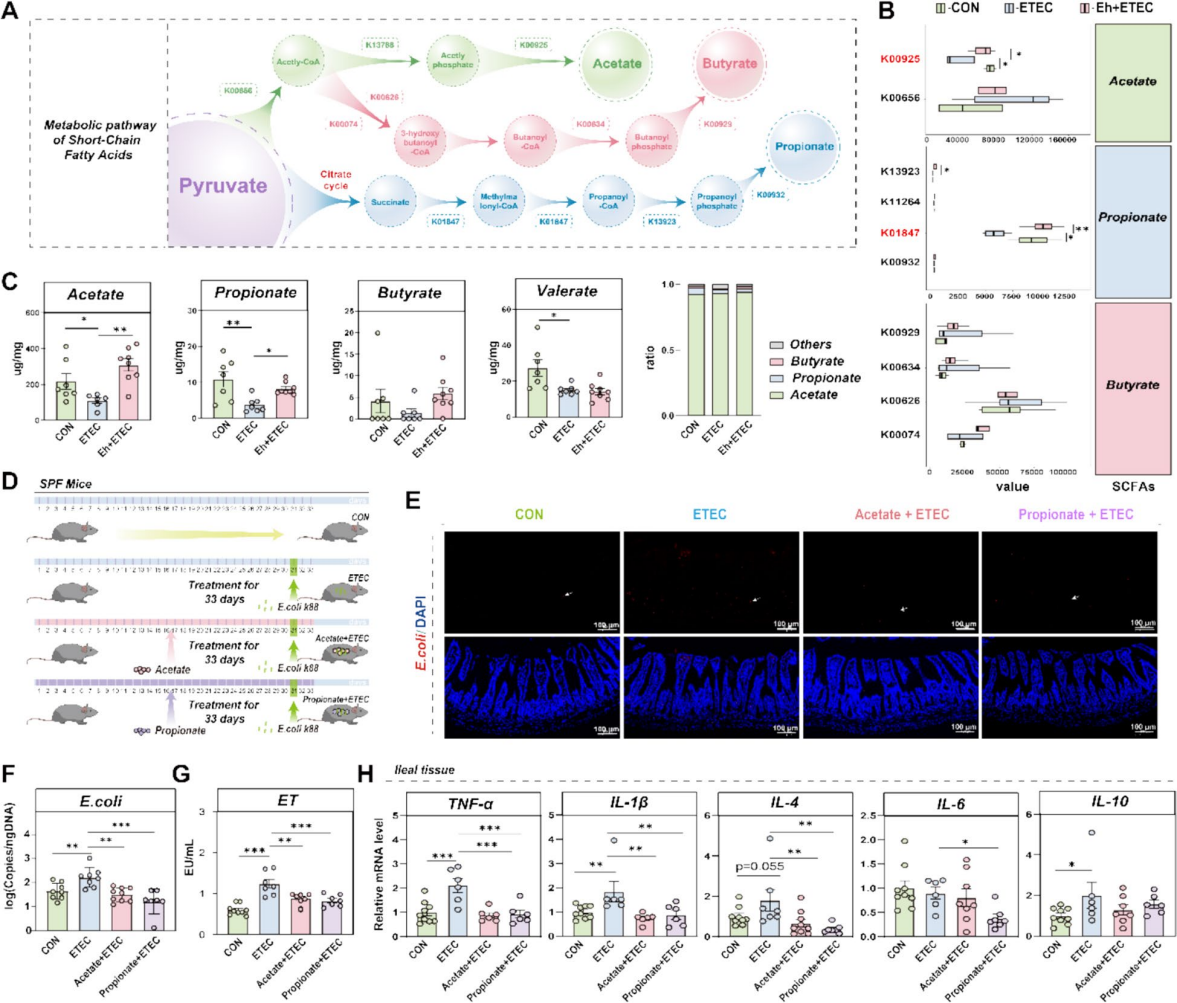
Acetate and propionate are the functional metabolites produced by *E. hirae*. **A** The metabolic pathways for synthesizing acetate, propionate, and butyrate from pyruvate, along with their corresponding KOs. **B** The relative expression of SCFAs metabolic pathway related KOs genes according bacterial function prediction. **C** The concentration of acetate, propionate, butyrate, valerate, and SCFAs ratio in ileal digesta of piglets. **D** Schematic diagram of oral acetate and propionate in mice. **E** FISH analysis of *E. coli* in the ileum of CON group, ETEC group, Acetate + ETEC group and Propionate + ETEC groups. Colonized *E. coli* were detected by FISH with Cy3-labeled, rRNA-targeted oligonucleotide probes. **F** The *E. coli* content in mice ileal digesta. **G** The concentration of ET in the mice serum samples. **H** The relative gene expression of TNF-α, IL-1β, IL-4, IL-6 and IL-10 in the ileal tissue

Changes in microbial community structure are often accompanied by alterations in metabolic function [41]. To explore the functional impact of *E. hirae* on gut microbiota, we analyzed the bacterial community’s functional capabilities using Phylogenetic Investigation of Communities by Reconstruction of Unobserved States (PICRUSt). In our results, we found that *E. coli* treatment significantly reduced the functional pathways related to pyruvate metabolism, while Ningxiang pig-derived *E. hirae* treatment significantly increased the enrichment of this pathway in the gut microbiota of *E. coli*-treated piglets (Figure S7A-B). Meanwhile, we analyzed the whole-genome sequencing and functional annotation of Ningxiang pig-derived *E. hirae*, and the results also revealed that *E. hirae* possesses a rich ability to utilize carbohydrates and may affect pyruvate metabolism (Figure S7A). The metabolic pathways for synthesizing acetate, propionate, and butyrate from pyruvate were shown in Fig. 3A. Compared to the CON group, ETEC treatment significantly reduced the expression of K00926 (acetate synthesis pathway) and K139923, K0184 (propionate synthesis pathway) genes in the gut microbiota (Fig. 3B). Notably, *E. hirae* significantly increased the expression of K00926 and K0184 genes involved in acetate and propionate synthesis, respectively (Fig. 3B), suggesting it has the potential to produce acetate and propionate. As expected, *E. coli* challenges significantly decreased the concentrations of acetate, propionate, and valerate, while oral administration of Ningxiang pig-derived *E. hirae* significantly increased the concentrations of acetate and propionate in *E. coli*-treated piglets (Fig. 3C). Consistent with the previous KOs results, butyrate was not significantly different between the three groups (Fig. 3B-C). Interestingly, we also found that acetate and propionate in the feces of Ningxiang piglets were significantly higher than those of Yorkshire piglets (Figure S8). These findings suggest that Ningxiang pig-derived *E. hirae* intervention can alter the microbial community structure of *E. coli* -treated piglets and is associated with an increased capacity for microbial interactions and the ability of the gut microbiota to produce acetate and propionate.

To further explore the role of acetate and propionate in modulating *E. coli*-induced intestinal inflammation, *E. coli*-infected mouse models were treated with these metabolites (Fig. 3D). The results showed that *E. coli* treatment facilitated its adhesion to the ileal tissue compared to the control group and increased the *E. coli* content in the ileal digesta (3E–F). At the same time, administration of acetate and propionate significantly reversed these changes in *E. coli*-infected mice, particularly propionate (3E–F). Additionally, acetate and propionate alleviated the morphological damage induced by *E. coli* and significantly reduced the levels of serum ET and DAO (Fig. 3G, Figure S9). Furthermore, acetate and propionate attenuated intestinal inflammation by decreasing pro-inflammatory cytokines TNF-α, IL-1β, IL-4, and IL-6 (Fig. 3H). Overall, these data are consistent with the hypothesis that acetate and propionate inhibit *E. coli* colonization and alleviate host inflammatory responses, with propionate exhibiting particularly strong effects.

### Acetate and propionate ameliorate intestinal inflammation by suppressing the Myd88-NF-κB pathway

**Fig. 4:**
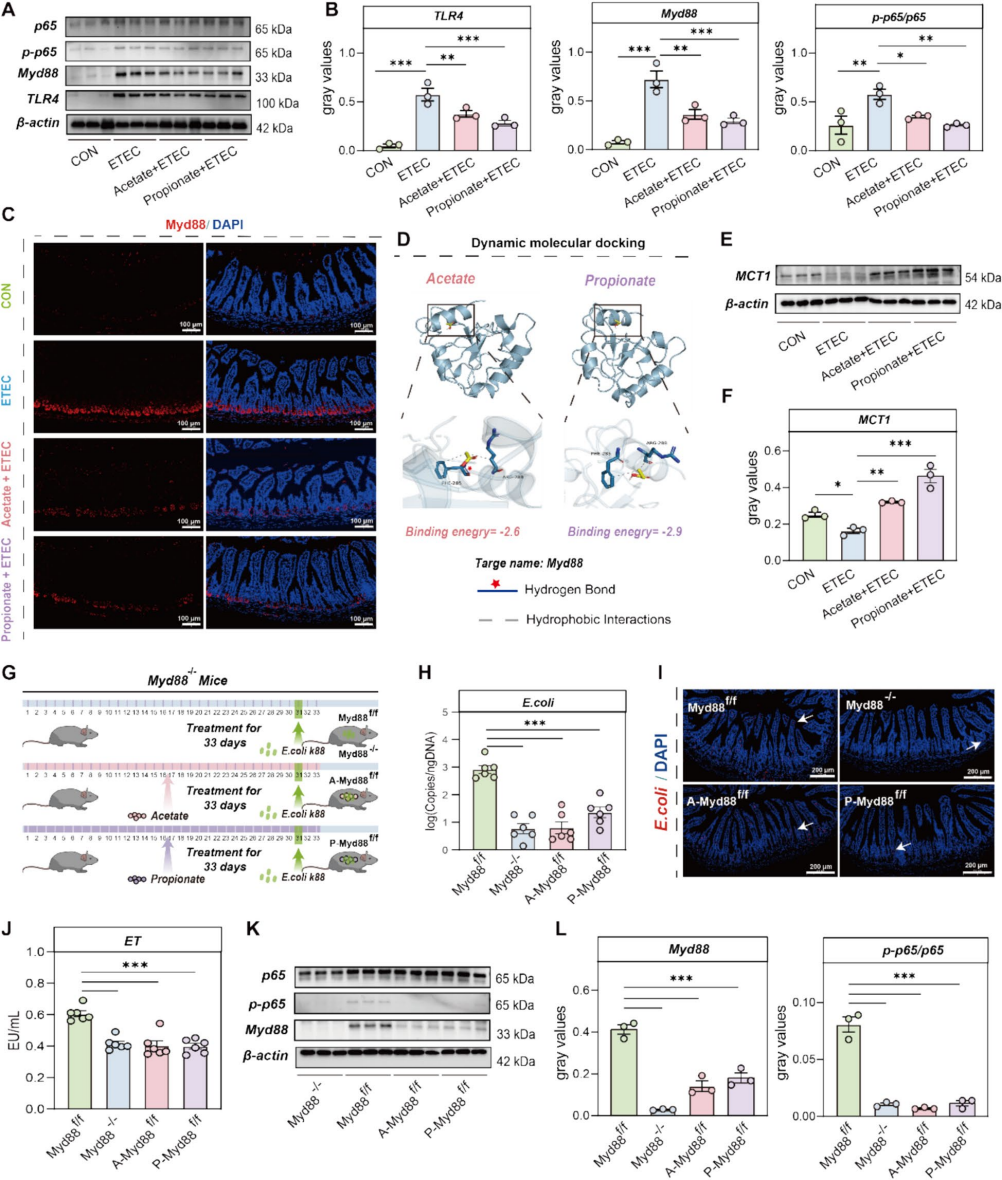
Acetate and propionate ameliorates intestinal inflammation by suppressing Myd88-NF-κB pathway. **A**-**B** Western blot images and relative densitometric analysis of mice ileal tissue TLR4, Myd88, and p-p65/p65 proteins. **C** Sections of the ileum are subjected to Myd88-immunofluorescence staining. Representative images of four groups are presented. **D** Dynamic molecular docking was employed to study interactions and binding affinity between acetate/propionate and Myd88, amino acid residues were predicted. **E**–**F** Western blot images and relative densitometric analysis of mice ileal tissue MCT1protein. **G** Schematic diagram of oral SCFAs in Myd88^f/f^/Myd88^ −/−^ mice. **H** The *E. coli* k88 content in mice ileal digesta. **I** FISH analysis of *E. coli* in the ileum of Myd88^f/f^, Myd88 ^−/−^, A-Myd88^f/f^ and P-Myd88^f/f^ mice. Colonized *E. coli* were detected by FISH with Cy3-labeled, rRNA-targeted oligonucleotide probes. **J** The concentration of ET in the mice serum samples. **K**-**L** Western blot images and relative densitometric analysis of ileal tissue Myd88, and p-p65/p65 proteins

Previous studies indicate that *E. coli* can bind to TLR4 ligands to exert its effects, subsequently activating the downstream Myd88-NF-κB pathway and inducing inflammatory responses [42, 43]. However, it remains uncertain whether *E. hirae* and key metabolites (acetate and propionate) can alleviate inflammation through this pathway. In our results, we found that *E. coli* treatment significantly increased the expression levels of TLR4, Myd88, and p65 in the ileal tissue, while Ningxiang pig-derived *E. hirae* treatment significantly decreased the expression of TLR4, Myd88, and p65 in *E. coli*-treated piglets (Figure S10A-C). The immunofluorescence results also showed that *E. coli* treatment significantly increased the expression of Myd88 + protein, while *E. hirae* treatment reversed this effect (Figure S10D). Subsequently, we used *E.coli* and Ningxiang pig-derived *E. hirae* to treat the IPEC-J2 cell. The results also showed that *E. hirae* significantly decreased the expression of TLR4, Myd88, and p65 (Figure S6E-F). Furthermore, consistent with our expectations, *E. coli* treatment significantly increased the gene expression of TLR4, Myd88, and P65 compared to the control group (Figure S11A-C), and elevated the expression levels of TLR4, Myd88, and p-p65/p65 proteins (Fig. 4A-B). However, acetate and propionate treatment effectively reversed these changes in *E. coli*-infected mice, particularly propionate (Fig. 4A-B, Figure S11A-C). Immunofluorescence staining also showed that *E. coli* treatment significantly increased Myd88 + protein expression in the ileal tissue, whereas acetate and propionate treatment reduced this expression (Fig. 4C). Similarly, we used *E.coli* and SCFAs to treat the IPEC-J2 cell. The results also showed that *E. coli* treatment significantly increased the expression of TLR4, Myd88, and P65 genes compared to the control group, while acetate and propionate treatment simultaneously reversed these changes (Figure S11D-E). Interestingly, dynamic docking analysis revealed a potential binding affinity between acetate, propionate, and Myd88 (Fig. 4D). Additionally, we identified a set of binding sites (i.e., PHE-285 and ARG-288) within a hydrophobic pocket responsible for the direct interaction of acetate and propionate with Myd88 (Fig. 4D). Notably, acetate formed a hydrogen bond with MyD88 at residue PHE-285 compared with propionate (Fig. 4D), suggesting that acetate may be more prone to bind directly to MyD88. However, acetate and propionate require transport via MCT-related proteins to enter the intestinal epithelial cells. Our results showed that acetate and propionate upregulated MCT1 protein expression in E. coli-treated mice, especially propionate (Fig. 4E-F). Meanwhile, Ningxiang pig-derived *E. hirae* treatment significantly increased the expression of SLC16A1 in E. coli-treated piglets (Figure S12).

Given that acetate and propionate activate MyD88 and its downstream NF-κB signaling, we generated Myd88 knockout (Myd88 ^−/−^) mice by crossing Myd88^f/f^ mice with Villin-Cre mice to create a model with epithelial cell-specific Myd88 deletion (Fig. 4G). The results showed that, compared to Myd88^f/f^ mice treated solely with *E. coli*, both Myd88 ^−/−^ mice treated with *E. coli* alone and Myd88^f/f^mice treated with *E. coli* plus acetate and propionate exhibited reduced *E. coli* adhesion in the ileal tissue and significantly lower *E. coli* content in the ileal digesta (F ig. 4H-I). Furthermore, compared to Myd88^f/f^ mice treated solely with *E. coli*, Myd88^−/−^ mice treated with *E. coli* and Myd88^f/f^ mice treated with *E. coli* plus acetate and propionate showed reduced intestinal morphological damage and significantly lower serum ET, DAO, and D-lactate levels (Fig. 4J, Figure S13). Additionally, the expression of inflammatory markers such as TNF-α, IL-1β, IL-4, and IL-6 in the ileal tissue was significantly reduced in both the Myd88^−/−^group, A-Myd88^f/f^ group, and P-Myd88^f/f^ group (Figure S14). Concurrently, compared to the Myd88^f/f^ group, the relative expression levels of Myd88 and its downstream p65 genes were also significantly lower in the Myd88^−/−^ group, A-Myd88^f/f^ group, and P-Myd88^f/f^group (Figure S15). Furthermore, Western blot analysis further showed that the absence of Myd88 in intestinal tissues indeed inhibited the downstream NF-κB signaling pathway (Fig. 4K-L). Moreover, acetate and propionate can achieve similar effects via the Myd88-NF-κB signaling pathway (Fig. 4K-L).

### SCFAs are downregulated in CD patients with Myd88-NF-κB pathway activation

**Fig. 5:**
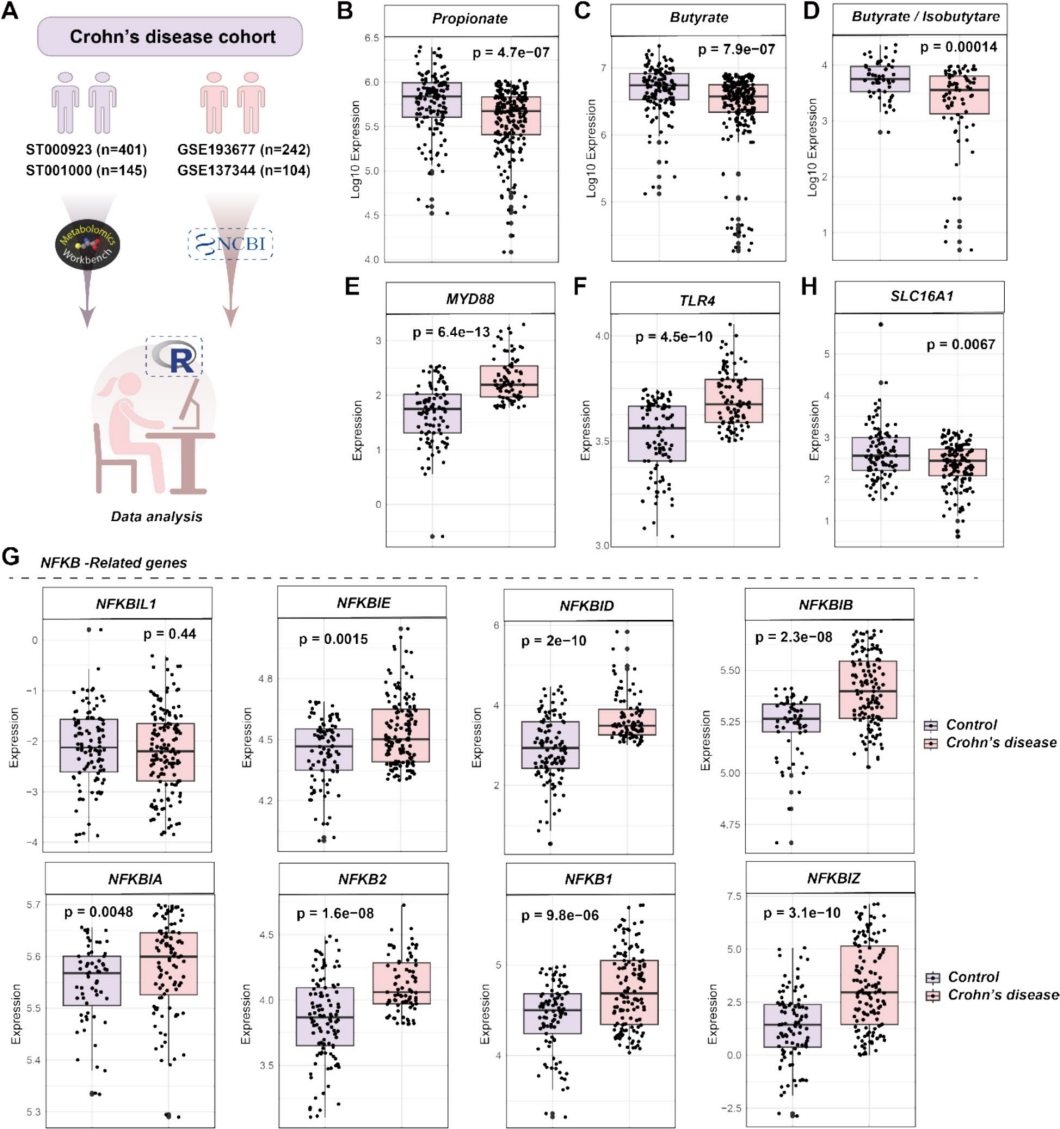
SCFAs are downregulated in Crohn’s disease patients with Myd88-NF-κB pathway activation. **A** Schematic representation of the Crohn’s disease cohort analyzed in the public database download. **B**-**D** Concentrations of SCFAs were showed as log10 (Peak Area) in Crohn’s disease cohort. **E**–**H** Bulk RNA-sequencing data for biopsies collected from ileum is accessed through NCBI database. Myd88, TLR4, SLC16A1 and NFκB-related genes expressions among Control and Crohn’s disease patients were shown.

To explore the clinical relevance of our findings, we retrieved and analyzed public datasets from Crohn’s disease (CD) cohorts. As CD primarily affects the terminal ileum [4], it is highly relevant to our study (Fig. 5A). Firstly, we found that the propionate, butyrate, and butyrate/isobutyrate concentrations in the feces were significantly reduced in CD patients by analyzing untargeted metabolic data from ST000923 and ST001000 in the Metabolomics Workbench (Fig. 5B-D). Furthermore, linear regression analyses revealed a significant negative correlation between the fecal concentrations of SCFAs (propionate, butyrate, and butyrate/isobutyrate) and the probability of CD occurrence (Figure S16), highlighting their importance as potential protective factors or predictive biomarkers for CD. Additionally, data from Takaishi et al. demonstrated that acetate in the feces was significantly reduced in CD patients (50.7 ± 14.3 mmol/L vs 28.7 ± 10.2 mmol/L; *p* < 0.01) [44].

To further validate our results, we retrieved and analyzed the bulk RNA-seq data from GSE193677 and GSE137344 involving the CD cohorts of biopsies collected from ileum. As expected, the expression of Myd88, TLR4, and NF-κB-related genes was significantly elevated in CD patients (5E–G), consistent with previous studies [45–47]. In addition, the expression of SLC16A1 was also markedly decreased in CD patients (Fig. 5H), which was consistent with a decrease in the concentration of SCFAs. The above results provide evidence for the clinical relevance of these metabolites in the pathogenesis of CD disease and suggest that the Myd88-NF-κB pathway may be a key target for alleviating intestinal inflammation in the host.

## Discussion

Bacterial infections, especially those caused by *E. coli*, play a critical role in disrupting gut homeostasis, leading to heightened immune activation, impaired barrier function, and subsequent intestinal inflammation [48, 49]. While antibiotics and anti-inflammatory drugs are commonly used to manage bacterial-induced intestinal inflammation, their long-term effectiveness is limited by recurrent infections, microbial resistance, and persistent dysbiosis [50, 51]. These challenges highlight the need for alternative therapeutic approaches that not only target pathogenic bacteria but also restore gut microbial balance and strengthen host defenses. In this study, we identified *E. hirae*, a potential probiotic strain isolated from the feces of Ningxiang piglets with low diarrhea incidence. Our findings suggest that Ningxiang pig-derived *E. hirae* effectively mitigates *E. coli* colonization, enhances microbial interactions, and produces key postbiotic metabolites—acetate and propionate—that contribute to gut homeostasis. By modulating the MyD88-NF-κB signaling pathway, Ningxiang pig-derived *E. hirae* reduced intestinal inflammation, strengthened gut barrier function, and improved host resistance to *E. coli*-induced enteritis. These results suggest that *E. hirae* supplementation or postbiotic application could serve as a promising strategy for managing bacterial-induced intestinal inflammation.

Although probiotics have emerged as a primary strategy for alleviating symptoms of IBDs, their underlying mechanisms of action remain to be fully elucidated [14, 16]. *E. hirae* is a LAB with significant probiotic potential, demonstrating diverse benefits in health management and disease treatment. Studies have shown that *E. hirae* enhances host immune function and modulates gut microbiota balance, promoting growth performance and improving disease resistance in animal models [52, 53]. Its bioactive components, such as bacteriocins and polysaccharides, exhibit antimicrobial activity while enhancing antioxidant capacity and regulating metabolism, thereby providing additional health benefits [54, 55]. Meanwhile, *E. hirae* has been shown to induce antitumor CD8 + T-cell responses, enhancing the efficacy of immune checkpoint inhibitors (e.g., anti-PD-1 antibodies) and chemotherapeutic agents (e.g., cyclophosphamide) [56]. Our previous study demonstrated that *E. hirae* isolated from Ningxiang pigs promotes gut development, strengthens intestinal barrier function, and improves microbial composition in weaned piglets [34]. The current study further provides evidence confirming that *E. hirae*, isolated from the feces of Ningxiang piglets with low diarrhea rates, effectively alleviates intestinal inflammation, particularly by inhibiting the colonization of *E. coli*, while revealing the critical role of gut microbiota in mediating inflammation.

Recent probiotic research has increasingly focused on postbiotic metabolites, which are bioactive compounds produced by beneficial microbes that exert health-promoting effects. Among these, SCFAs such as acetate and propionate are well recognized for their anti-inflammatory and immunoregulatory properties [20–22]. In this study, genomic sequencing revealed that Ningxiang pig-derived *E. hirae* harbors genes involved in pyruvate metabolism, suggesting its potential to enhance SCFA production. In the *E. coli*-challenged piglet model, acetate and propionate levels were significantly reduced, whereas butyrate levels remained unchanged. This indicates that *E. coli* infection may contribute to inflammation by specifically disrupting acetate and propionate metabolism, rather than butyrate metabolism. Supplementation with Ningxiang pig-derived *E. hirae* restored acetate and propionate levels, supporting their role in mitigating inflammation. Further validation in *E. coli*-treated mouse and IPEC-J2 cell models demonstrated that acetate and propionate supplementation effectively reduced inflammatory cytokine levels and key serum biomarkers such as D-lactate, ET, and DAO, thereby alleviating inflammation. Notably, despite butyrate’s well-documented role in intestinal health [57–59], our results suggest that acetate and propionate play a more dominant role in modulating inflammation under *E. coli* infection. This raises the possibility of an alternative regulatory mechanism by which probiotics influence host immunity and gut homeostasis. Furthermore, analysis of publicly available metabolomics data confirmed a reduction in SCFAs, particularly acetate and propionate, in the feces of CD patients, consistent with our experimental results. This reduction in SCFAs was associated with gut microbiota dysbiosis, marked by a decline in SCFA-producing bacteria. The restoration of acetate and propionate levels through probiotics such as *E. hirae* could therefore represent a novel strategy to rebalance the gut microbiome and alleviate symptoms of IBDs. This underscores the potential clinical relevance of our findings and supports exploring SCFA-based therapies to restore gut homeostasis and reduce inflammation in patients with *E. coli*-induced enteritis and CD.

*E. coli* infection initiates a pro-inflammatory response by activating the NF-κB signaling pathway, which plays a central role in immune system homeostasis and activation [60, 61]. When *E. coli* proliferates in large numbers, it releases ET, which bind to the pattern recognition receptor TLR4 in intestinal epithelial cells, thereby activating the downstream TLR4/MyD88/NF-κB signaling pathway and triggering inflammation [62]. Our research indicated that the TLR4-MyD88-NF-κB pathway was significantly activated in all *E. coli* infection models (pigs, mice, and cells), whereas supplementation with Ningxiang pig-derived *E. hirae* or SCFAs (acetate and propionate) partially reversed this activation. Interestingly, the bulk RNA-seq data from public datasets of CD also exhibited significant activation of this pathway, underscoring the potential role of the MyD88-NF-κB pathway in the progression of IBD. Additionally, dynamic molecular docking analysis suggested that SCFAs may directly interact with MyD88. Monocarboxylate transporter 1 (MCT1), a transporter protein located on the cell membrane, facilitates the transport of SCFAs and other monocarboxylates into cells [63]. In our results, *E. coli* infection significantly reduced MCT1 expression, whereas SCFA supplementation restored MCT1 protein levels. We hypothesize that SCFAs are transported into cells via MCT1 and subsequently may bind to MyD88, contributing to their anti-inflammatory effects. Meanwhile, gavage with the probiotic strain *E. hirae* significantly upregulated the expression of the SLC16A1 gene (encoding MCT1) [64] in the ileum of ETEC-challenged piglets. To further validate our results, we retrieved and analyzed the SLC16A1 gene from the bulk RNA-seq data from public datasets of CD. Consistently, the SLC16A1 gene in the ileum of CD patients was significantly lower than those of healthy volunteers. These findings align with the observed increase in SCFA levels in the piglets and CD patients.

Collectively, our results demonstrate that *E. hirae* acts against *E. coli*-induced intestinal inflammation through the acetate/propionate-MyD88-NF-κB pathway, highlighting the therapeutic potential of SCFA-mediated innate immunity modulation for bacterial inflammation. However, several limitations of this study should be considered. First, while our multi-omics approach and animal models provide supportive evidence for the acetate/propionate-MyD88-NF-κB axis, the precise molecular details of how SCFAs modulate MyD88 signaling in vivo require further validation. Second, the protective effects observed may involve additional microbial metabolites and pathways beyond those examined here. Third, translating these findings from animal models to human applications requires careful consideration of physiological differences.

## Conclusion

**Fig. 6:**
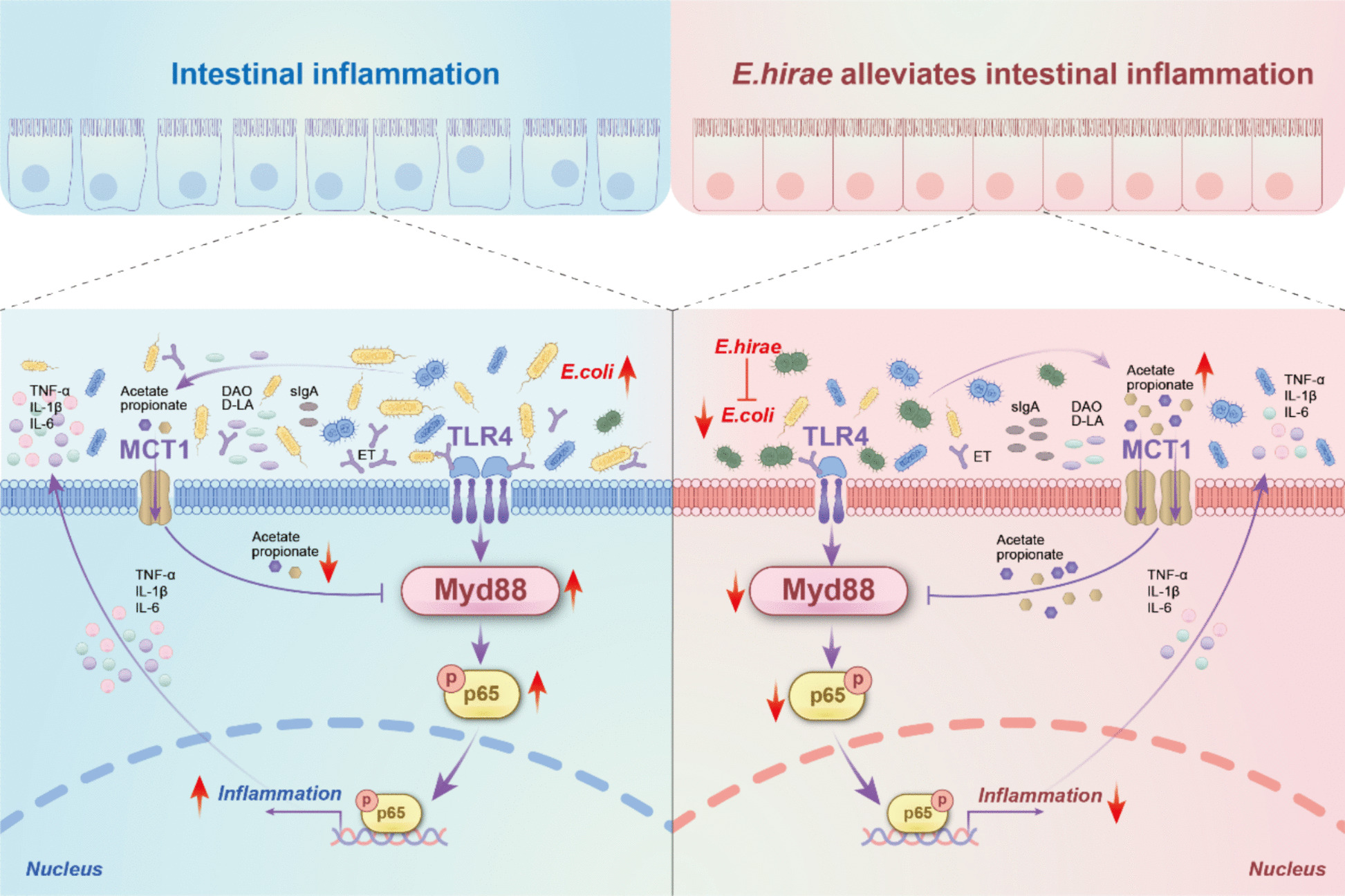
Schematic diagram of *E. hirae* and its postbiotics in addressing *E. coli*-associated intestinal inflammation

In summary, our findings demonstrate that Ningxiang piglet-derived *E. hirae* mitigates *E. coli*-induced gut dysbiosis and inflammation by restoring microbial balance, elevating acetate/propionate production, and suppressing the MyD88-NF-κB pathway (Fig. 6). The correlation between reduced SCFAs and MyD88-NF-κB activation in CD patients further supports the clinical relevance of this axis. Our findings provide a theoretical basis for further research into probiotics or SCFA-targeted therapies for bacterial enteritis.

## Supplementary Information

Supplementary Material 1.

Supplementary Material 2.

## Data Availability

No datasets were generated or analysed during the current study.
