# Supplementary material for: Ningxiang pig-derived Enterococcus hirae protects against E. coli-induced gut dysbiosis and inflammation via acetate/propionate-MyD88-NF-κB axis in piglets
Source: Microbiome. 2026 Jan 6;14:55. doi: 10.1186/s40168-025-02310-8 (PMC12871038; doi:10.1186/s40168-025-02310-8)
Supplement: Supplementary file 2 — Supplementary Material 1. [file 40168_2025_2310_MOESM1_ESM.docx]

**Supplementary Figure**


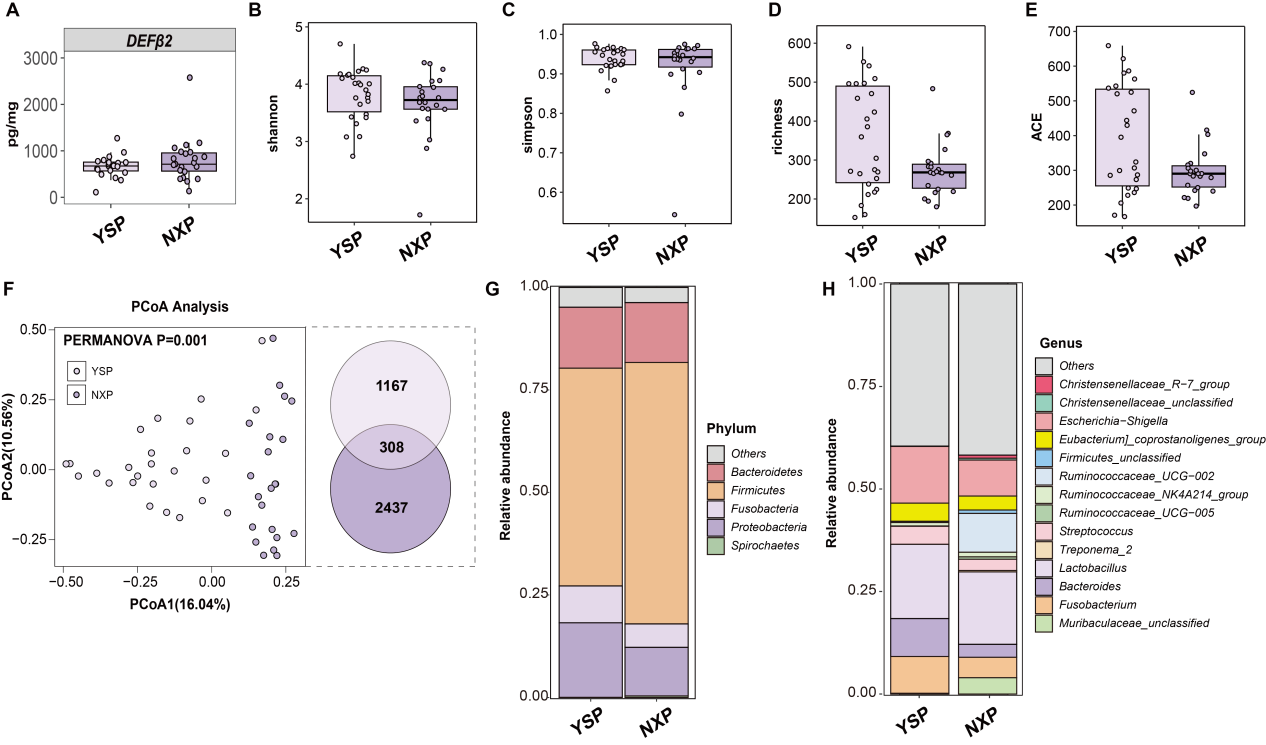


**Figure S1. Differential Incidence of Gut Microbial Characteristics between Ningxiang and Yorkshire Piglets.** (A) The result of DEFβ2 concentration in two breeds piglets stool samples at day-7. (B-E) α-diversity (Shannon index, simpson index, richness index and ACE diversity index) between Ningxiang and Yorkshire piglets. (F) Principal coordinate analysis (PCoA) of Ningxiang piglets and Yorkshire piglets samples based on Bray-Curtis dissimilarity. (G-H) Comparisons of the relative abundance of gut phylum and genus among two breeds. The mean relative abundance of piglets was sorted, and only the top 5 phylum and the top 15 genus are plotted, with the rest going to “others”.


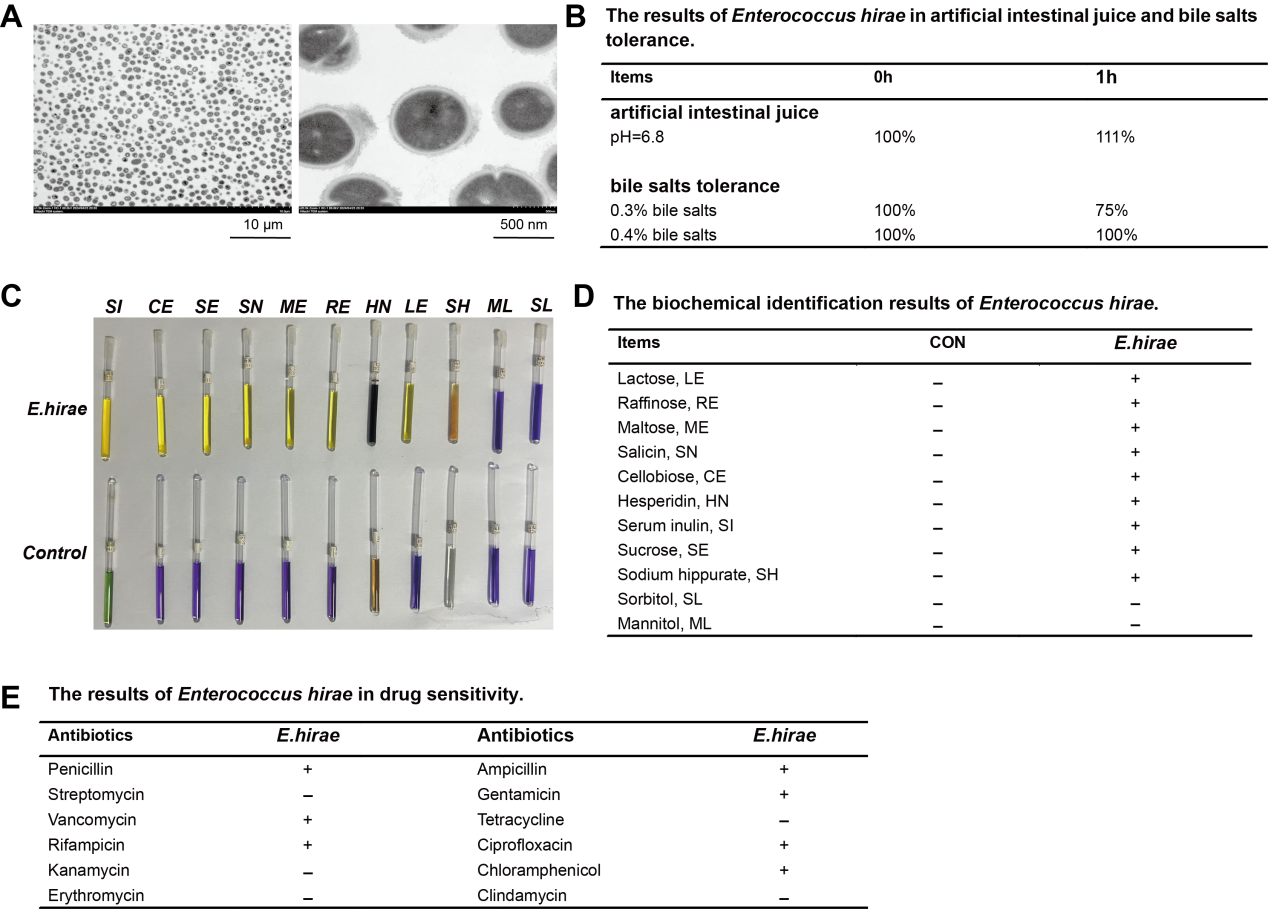


**Figure S2. In vitro probiotic effect of *E.hirae*.** (A) Transmission electron microscopy of *E.hirae*. (B) The results of *E.hirae* in artificial intestinal juice and bile salts tolerance. (C-D) The biochemical identification results of *E.hirae*. (E) The results of *E.hirae* in drug sensitivity.


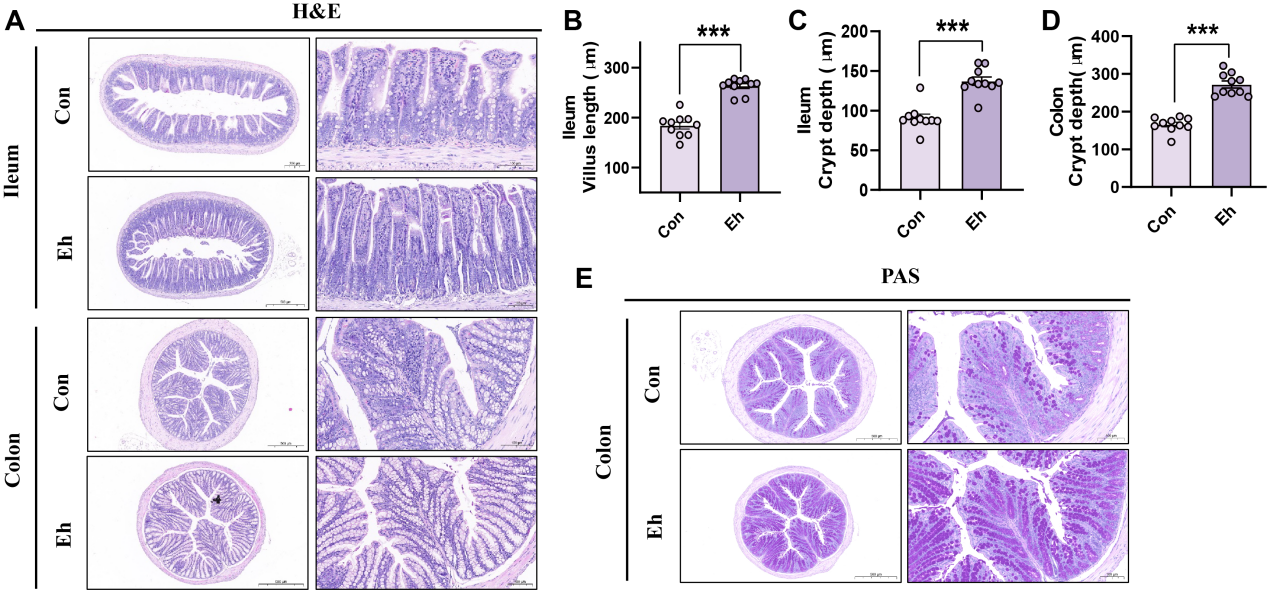


**Figure S3. In vivo probiotic effect of *E.hirae* by using a mouse model.** (A) The morphology images of the ileum and colon. (B-D) The villus length, crypt depth of ileum, and crypt depth of colon. (E) PAS staining for mucus layer and mucus-producing goblet cells in colon.


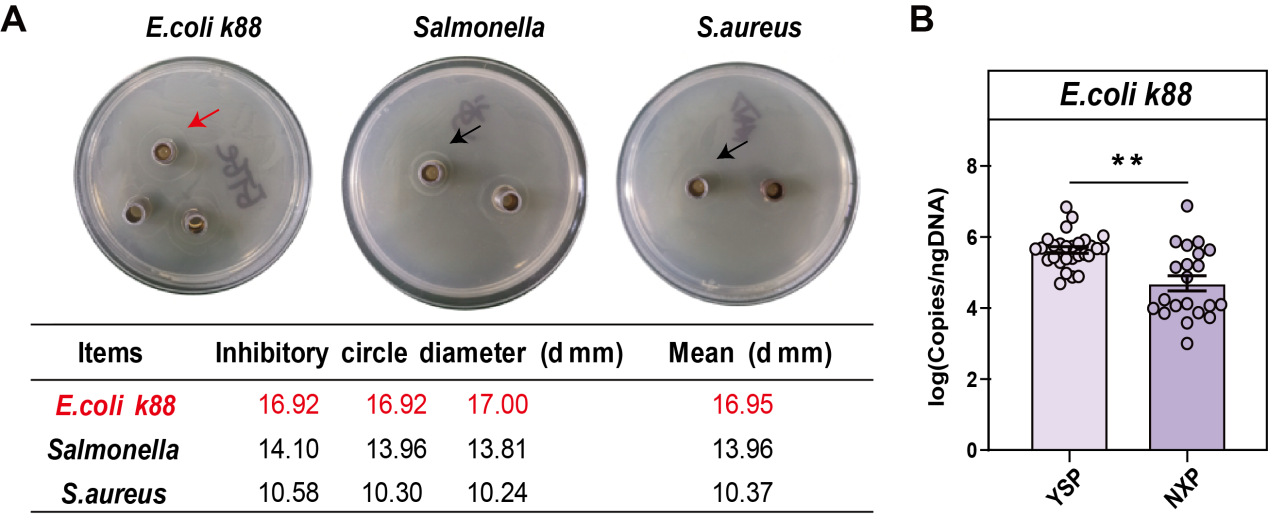


**Figure S4. *E.hirae* inhibit a variety of pathogens in vitro.** (A) In vitro antimicrobial test results of *E.hirae* strains. (B) The fecal *E. coli* content in the Yorkshire piglets and Ningxiang piglets.


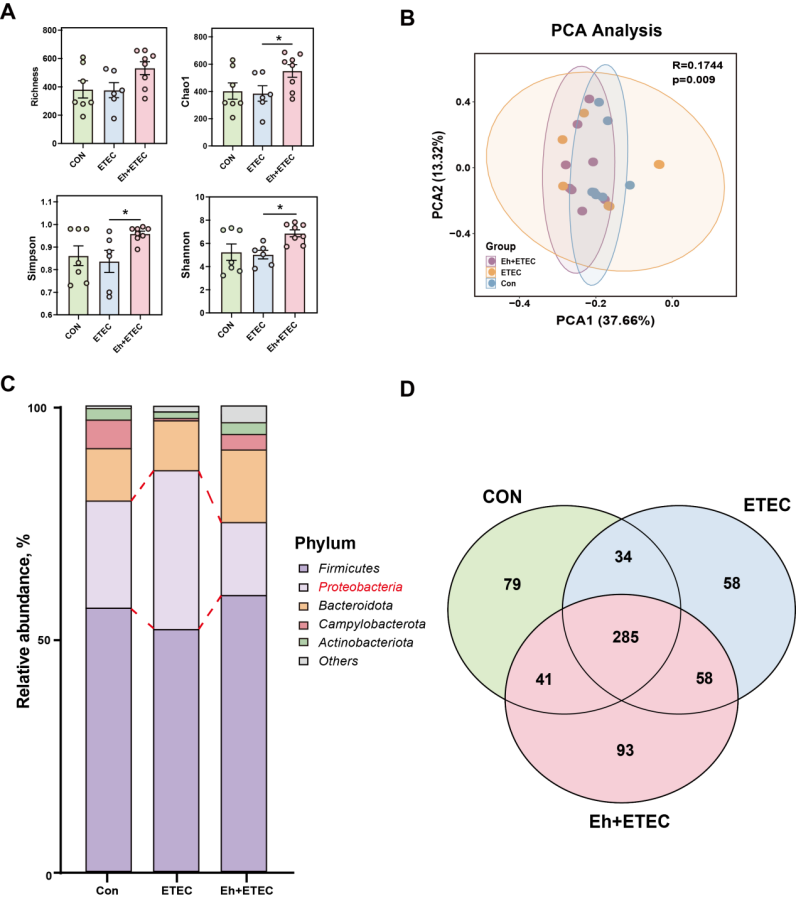


**Figure S5. Effects of *E.hirae* on gut microbiota of ETEC-challenged piglets.** (A) α-diversity (Shannon index, simpson index, richness index and ACE diversity index) between CON group, ETEC group and Eh+ETEC groups. (B) Principal coordinate analysis (PCoA) of ileum mucosal samples based on Bray-Curtis dissimilarity. (C) Comparisons of the relative abundance of gut phylum among CON group, ETEC group and Eh+ETEC groups. The mean relative abundance of piglets was sorted, and only the top 5 phylum and the top 15 genus are plotted, with the rest going to “Others”. (D) The result of bacteria ASVs between CON group, ETEC group and Eh+ETEC groups.


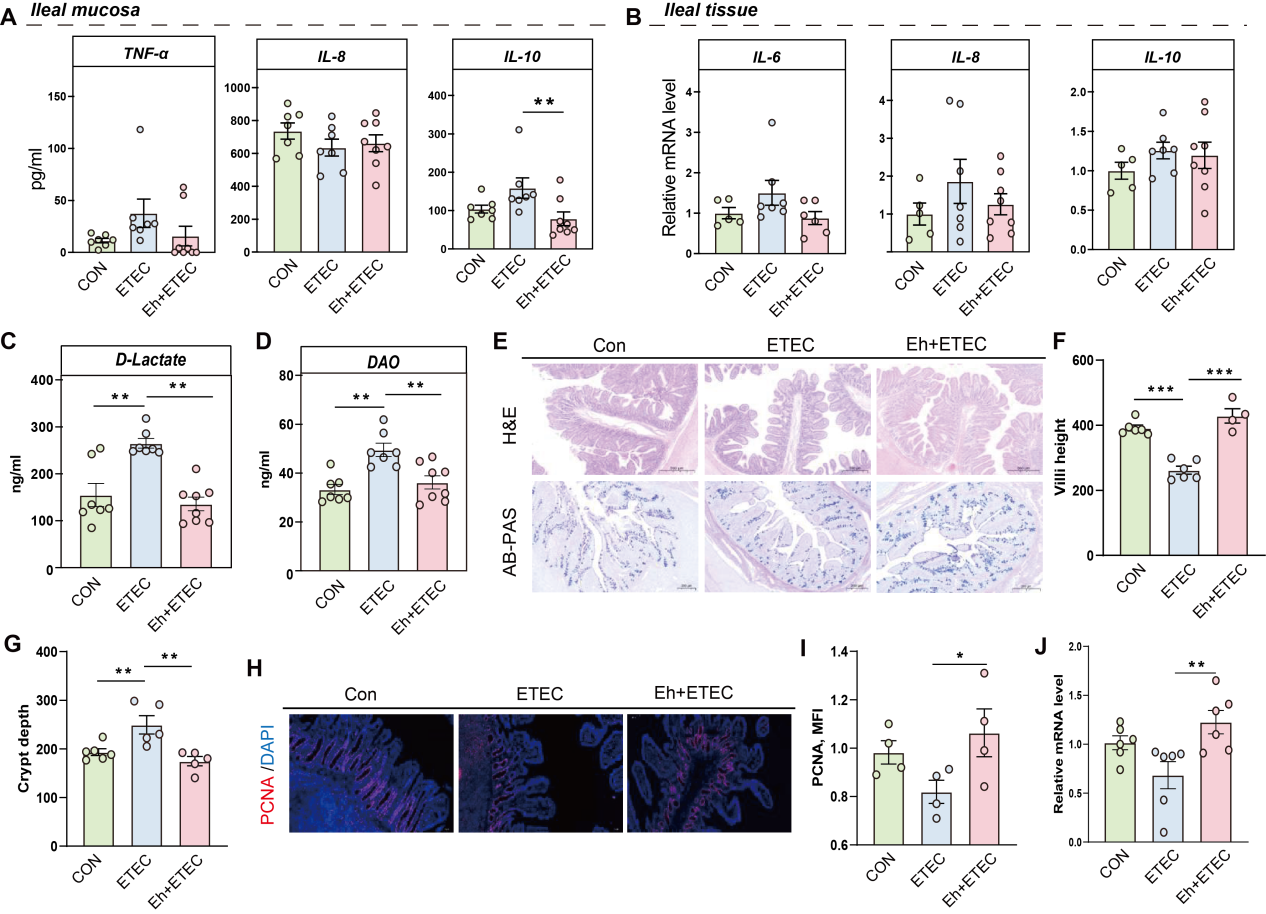


**Figure S6. Effects of *E.hirae* on intestinal inflammation and intestinal damage of ETEC-challenged piglets.** (A) The concentration of TNF-α, IL-8 and IL-10 in the ileal mucosa. (B) The relative gene expression of IL-6, IL-8 and IL-10 in the ileal tissue. (C-D) The concentration of DAO (C) and D-lactate (D) in the piglet serum samples. (E) The intestinal morphology and AB-PAS staining images of the ileum. (F-G) The villus length, crypt depth of ileum. (H-I) Sections of the ileum are subjected to PCNA-immunofluorescence staining. Representative images and statistical results of protein expression are presented. (J) The relative protein expression levels of PCNA in ileum.


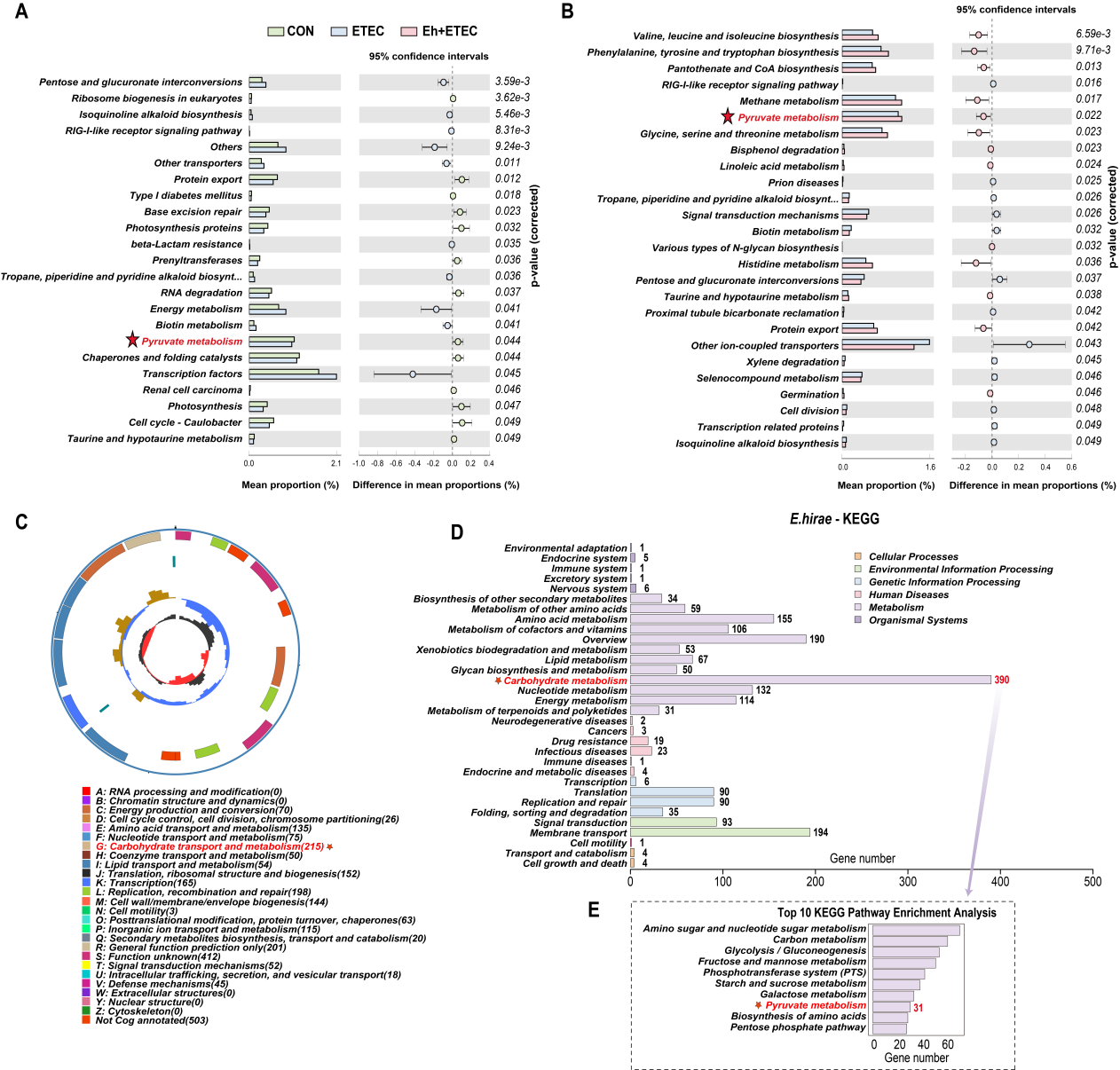


**Figure S7. Functional Characterization of *E.hirae*.** (A-B) The functional capabilities of the bacterial communities among CON group, ETEC group and Eh+ETEC groups. (C) The whole-genome sequencing and functional annotation of Ningxiang pig-derived *E.hirae*. (D-E) The KEGG pathway enrichment analysis of Ningxiang pig-derived *E.hirae* genome.


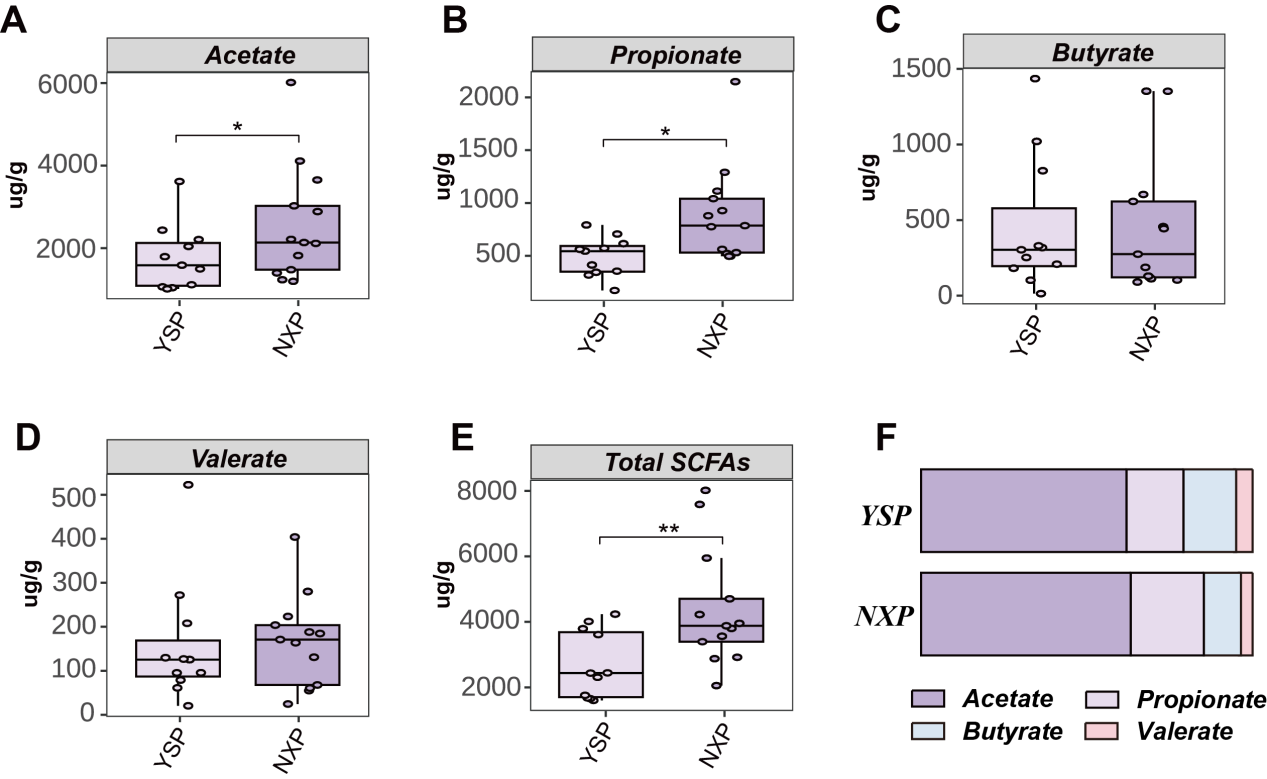


**Figure S8. The concentration of acetate, propionate, butyrate, valerate, and SCFAs ratio in faeces of Ningxiang piglets and Yorkshire piglets.**


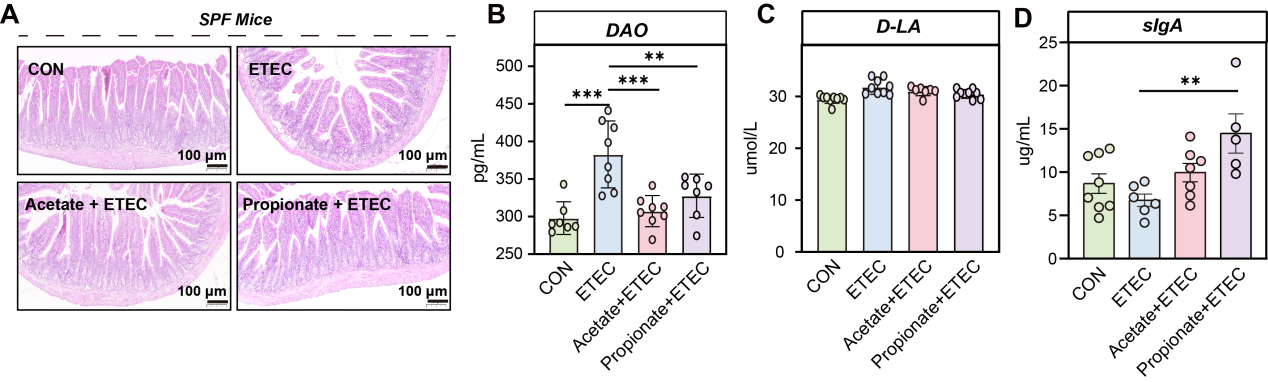


**Figure S9. Effects of acetate and propionate on intestinal health of ETEC-challenged mice.** (A) The intestinal morphology images of the ileum. (B-D) The concentration of DAO (B), D-lactate (C) and sIgA (D) in the mice serum samples.

**
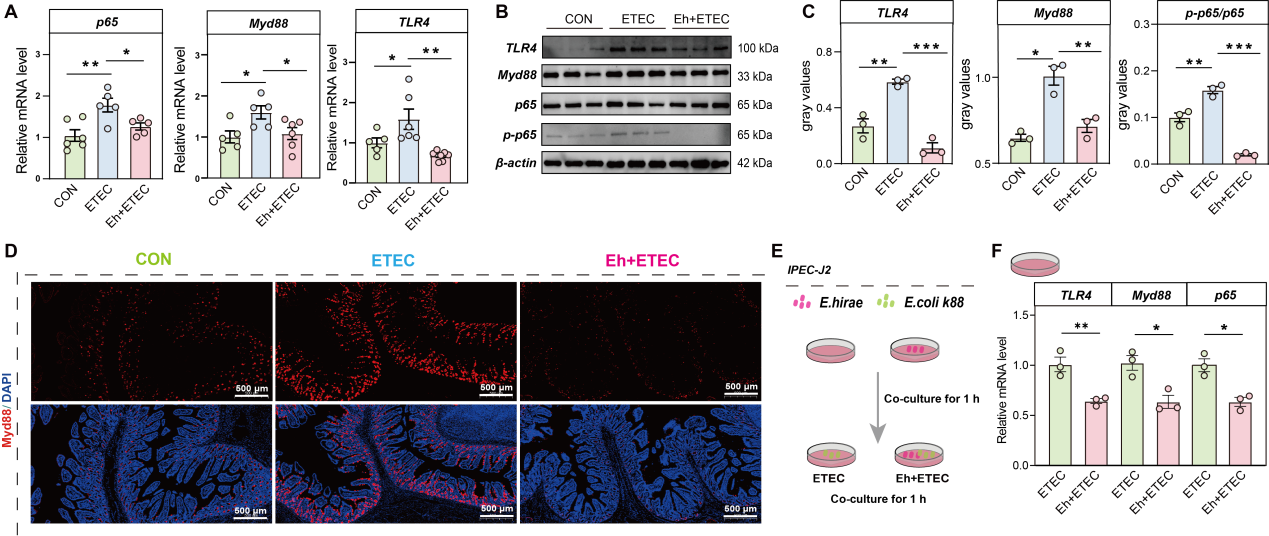
Figure S10. Effects of *E.hirae* on TLR4-Myd88-NF-κB pathway of ETEC-challenged piglets.** (A) The relative expression of TLR4, Myd88, p65 genes in the piglet ileal tissue. (B) Western blot images of ileal tissue TLR4, Myd88, p65, p-p65 and β-actin proteins. (C) Relative densitometric analysis of ileal tissue TLR4, Myd88, p-p65/p65. (D) Sections of the ileum are subjected to Myd88-immunofluorescence staining. Representative images of CON group, ETEC group, Acetate+ETEC group and Propionate+ETEC groups are presented. (E) Schematic diagram of the IPEC-J2 cell experiment. (F) The relative expression of TLR4, Myd88, p65 genes in the IPEC-J2 cell.


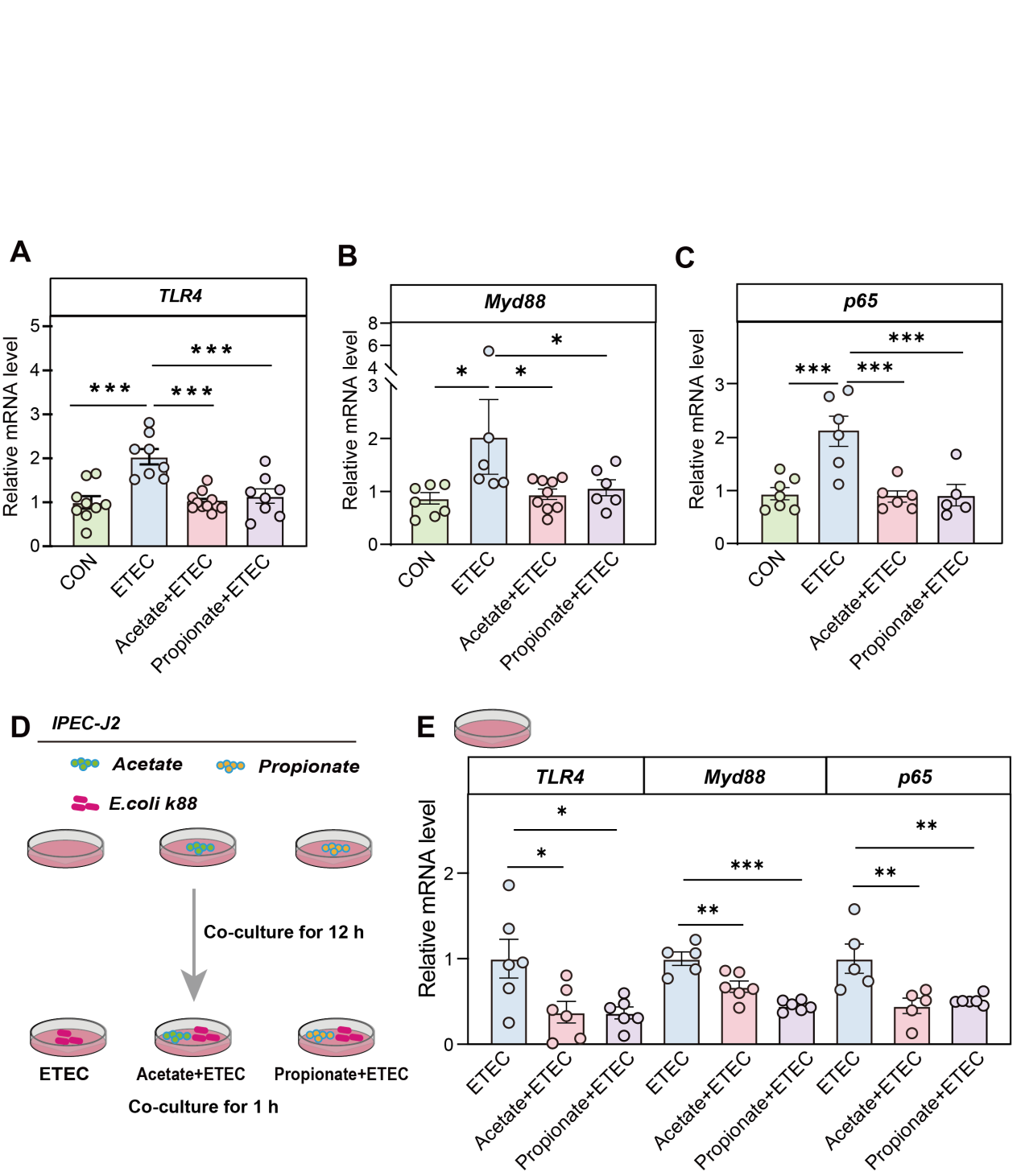


**Figure S11. Effects of acetate and propionate on TLR4-Myd88-NF-κB pathway of ETEC-challenged mice and IPEC-J2 cells.** (A-C) The relative expression of TLR4, Myd88, p65 genes in the mice ileal tissue. (D) Schematic diagram of the IPEC-J2 cell experiment. (E) The relative expression of TLR4, Myd88, p65 genes in the IPEC-J2 cell.


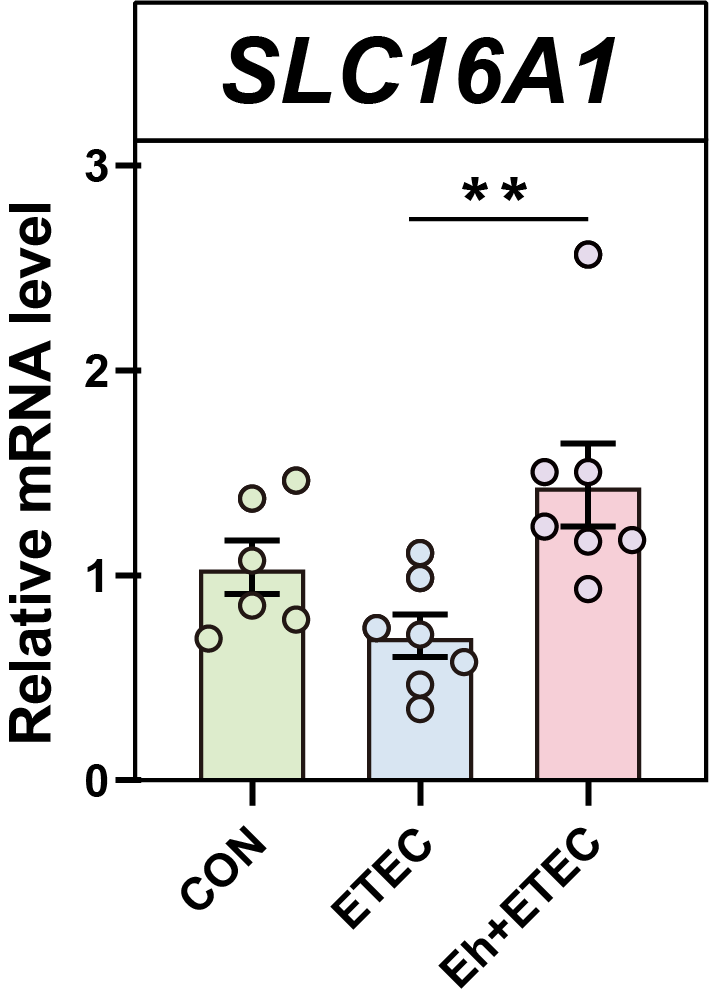


**Figure S12.** **The relative expression of SLC16A1 genes in the piglets ileal tissue.**


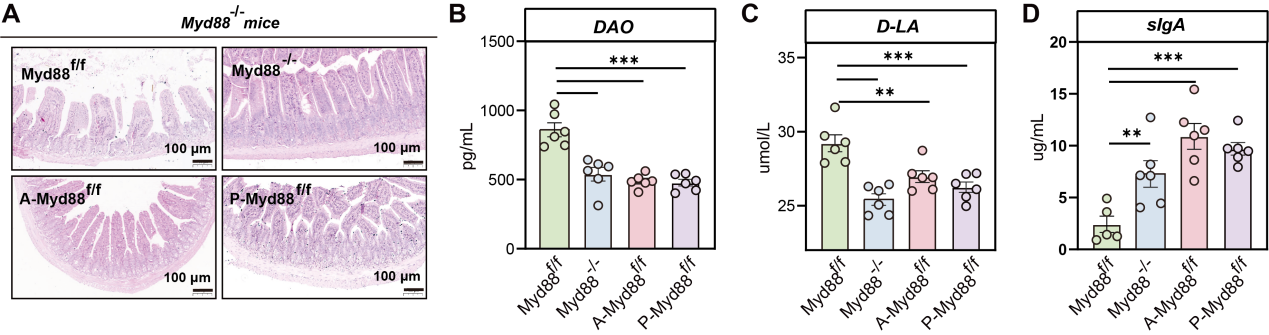


**Figure S13. Effects of acetate and propionate on intestinal health of ETEC-challenged Myd88^-/-^mice.** (A) The intestinal morphology images of the ileum. (B-D) The concentration of DAO (B), D-lactate (C) and sIgA (D) in the mice serum samples.


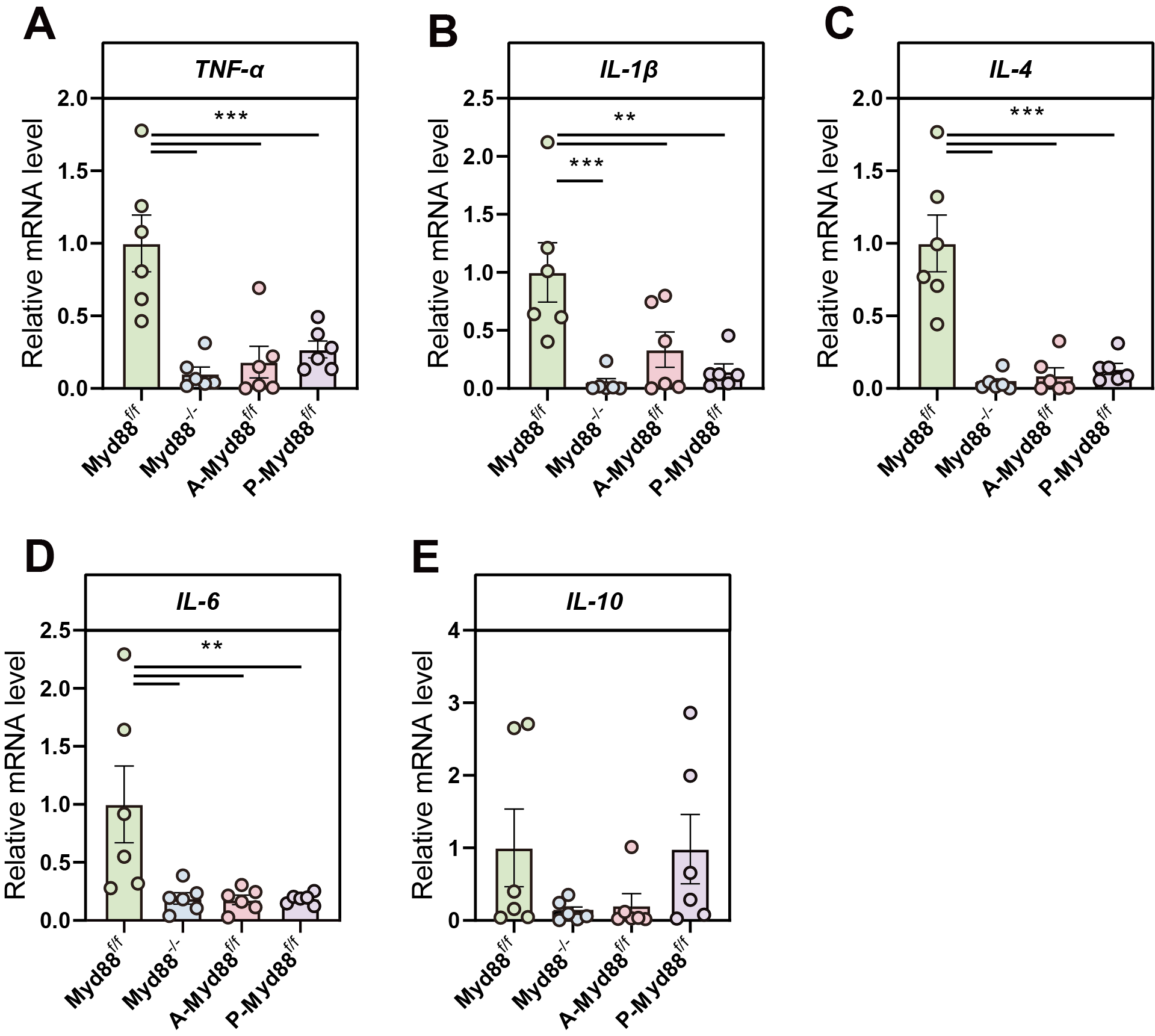


**Figure S14. Effects of acetate and propionate on intestinal inflammatory of ETEC-challenged Myd88^-/-^ mice.** (A-E) The relative gene expression of inflammatory and immune regulatory factors in the mice ileal tissue.


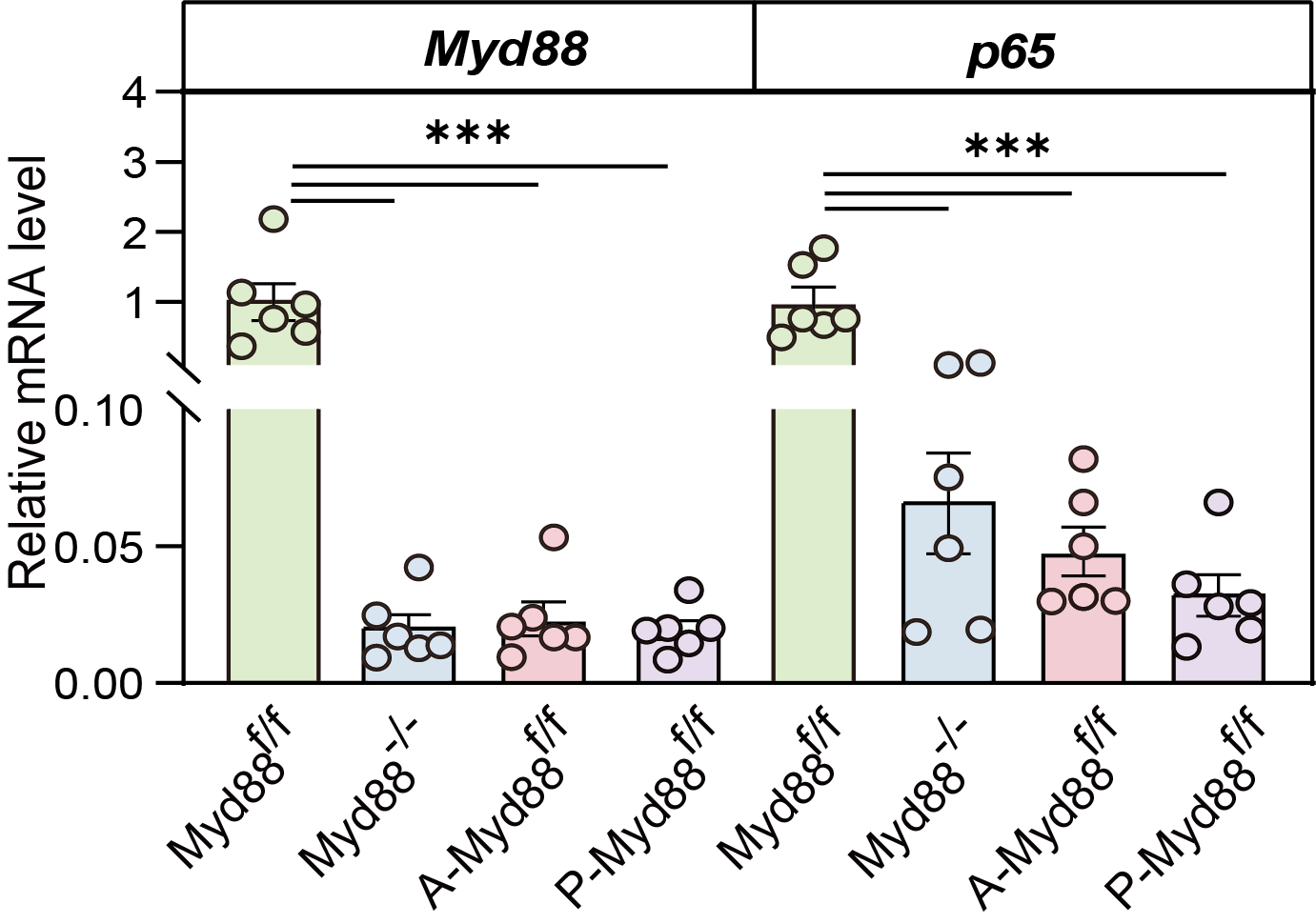


**Figure S15. Effects of acetate and propionate on TLR4-Myd88-NFKB pathway of ETEC-challenged Myd88^-/-^ mice.** The relative expression of Myd88, p65 genes in the mice ileal tissue.


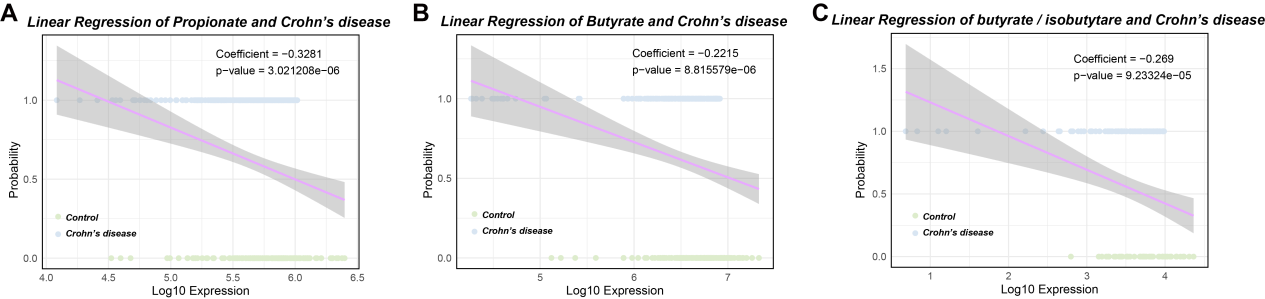


**Figure S16. Linear regression analyses of SCFAs (Propionate, Butyrate, and Butyrate/Isobutyrate) and the probability of Crohn's disease occurrence.** The analysis was conducted by assigning Crohn's disease patients a value of 1 and control subjects a value of 0 as the dependent variable. Linear regression models were then applied to evaluate the association between the log-transformed fecal concentrations of SCFAs (Propionate (A), Butyrate (B), and Butyrate / Isobutyrate (C)) and the probability of Crohn's disease occurrence.
