# Supplementary material for: Ningxiang pig-derived Enterococcus hirae protects against E. coli-induced gut dysbiosis and inflammation via acetate/propionate-MyD88-NF-κB axis in piglets
Source: Microbiome. 2026 Jan 6;14:55. doi: 10.1186/s40168-025-02310-8 (PMC12871038; doi:10.1186/s40168-025-02310-8)
Supplement: Supplementary file 3 — Supplementary Material 2. [file 40168_2025_2310_MOESM2_ESM.docx]

**Supplementary Table**

**Supplementary TableS1. Primers used for gene expression analysis through Real-Time PCR.**

| **Genes** | **Forward** | **Reverse** |
| --- | --- | --- |
| **Pig** |  |  |
| *PCNA* | TACGCTAAGGGCAGAAGATAATG | CTGAGATCTCGGCATATACGTG |
| *IL-6* | ATCCTCGACGGCATCTC | TCAGCCATCTTTGGAAGG |
| *TNF-α* | TGTGTGGCTGCAGGAAGAAC | GCAATTGAAGCACTGGAAAAGG |
| *IL-8* | GACATACTCCAAACCTTTCCA | AACTTCTCCACAACCCTCTG |
| *IL-10* | CTGCATCCACTTCCCAACCA | AGAAACTCTTCACTGGGCCG |
| *IL-1β* | AACGTGCAGTCTATGGAGT | GAACACCACTTCTCTCTTCA |
| *Myd88* | GTGCCGTCGGATGGTAGTG | TCTGGAAGTCACATTCCTTGCTT |
| *TLR4* | TGGATTTATCCAGCCAGGACGA | TGTATGAAGTGCTGGGACACC |
| *P65* | AGCCATTGACGTGATCCAGG | CGAAATCGTGGGGCACTTTG |
| *SLC16A1* | ACATGGTAGCCAGACCCTCT | GGCTTCTCAGCAGCGTCTAT |
| *β-actin* | GCGTAGCATTTGCTGCATGA | GCGTGTGTGTAACTAGGGGT |
| ***Cell*** |  |  |
| *Myd88* | GTGCCGTCGGATGGTAGTG | TCTGGAAGTCACATTCCTTGCTT |
| *TLR4* | TGGATTTATCCAGCCAGGACGA | TGTATGAAGTGCTGGGACACC |
| *P65* | AGCCATTGACGTGATCCAGG | CGAAATCGTGGGGCACTTTG |
| *β-actin* | GTCCACCTTCCAGCAGATGT | GAAAGGGTGTAAAACGCAGC |
| ***Mice*** |  |  |
| *IL-6* | CTGCAAGAGACTTCCATCCAG | AGTGGTATAGACAGGTCTGTTGG |
| *TNF-α* | CAGGCGGTGCCTATGTCTC | CGATCACCCCGAAGTTCAGTAG |
| *IL-4* | GGTCTCAACCCCCAGCTAGT | GCCGATGATCTCTCTCAAGTGAT |
| *IL-10* | AGGCGCTGTCATCGATTTCT | GGCCTTGTAGACACCTTGGTC |
| *IL-1β* | TTCAGGCAGGCAGTATCACTC | GAAGGTCCACGGGAAAGACAC |
| *Myd88* | CCGCCTATCGCTGTTCTTGA | CTGCCAGGCATCCAACAAAC |
| *TLR4* | AGATCTGAGCTTCAACCCCTTG | GCAGAAACATTCGCCAAGCA |
| *P65* | GCAGGAACTCAAGGGAGCTAA | CGTTGTTGTCCAGGACCAGT |
| *β-actin* | CACTGTCGAGTCGCGTCC | TCATCCATGGCGAACTGGTG |
| **Bacteria** |  |  |
| *E.coli* | CATGCCGCGTGTATGAAGAA | CGGGTAACGTCAATGAGCAAA |
| *413bp* | ATCTTTTACTTTCACCAGCGTTT | CACTCAACGCCTATCTCGGTCT |
| *96bp* | ATCTTTTACTTTCACCAGCGTTT | AGTATTCAAGCATTTCCGTGTCG |
